# Halting the Spread of Herpes Simplex Virus-1: The Discovery of an Effective Dual αvβ6/αvβ8 Integrin Ligand

**DOI:** 10.1021/acs.jmedchem.1c00533

**Published:** 2021-05-07

**Authors:** Stefano Tomassi, Vincenzo Maria D’Amore, Francesco Saverio Di Leva, Andrea Vannini, Giacomo Quilici, Michael Weinmüller, Florian Reichart, Jussara Amato, Barbara Romano, Angelo Antonio Izzo, Salvatore Di Maro, Ettore Novellino, Giovanna Musco, Tatiana Gianni, Horst Kessler, Luciana Marinelli

**Affiliations:** † Dipartimento di Farmacia, Università degli Studi di Napoli “Federico II”, Via D. Montesano 49, 80131 Naples, Italy; ‡ Department of Experimental, Diagnostic and Specialty Medicine, University of Bologna, 40126 Bologna, Italy; § Biomolecular NMR Unit c/o IRCCS S. Raffaele, Via Olgettina 58, 20132 Milano, Italy; ∥ Institute for Advanced Study, Department of Chemistry, Technische Universität München, Lichtenbergstraße 4, 85748 Garching, Germany; ⊥ DiSTABiF, University of Campania “Luigi Vanvitelli”, Via Vivaldi 43, 81100 Caserta, Italy; # Facoltà di Medicina e Chirurgia, Università Cattolica del Sacro Cuore, Largo Francesco Vito, 1, 00168 Roma, Italy

## Abstract

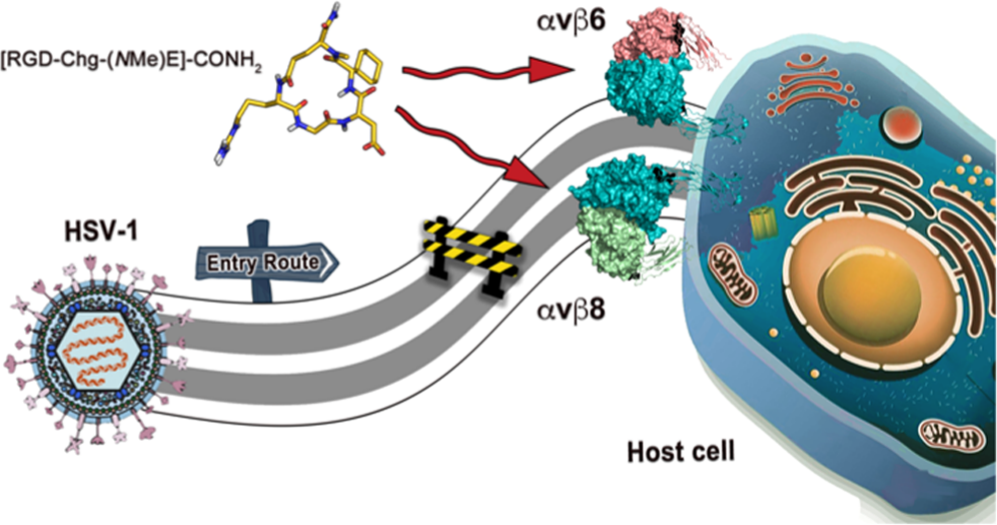

Over recent years, αvβ6 and αvβ8 Arg-Gly-Asp (RGD) integrins have risen to prominence as interchangeable co-receptors for the cellular entry of herpes simplex virus-1 (HSV-1). In fact, the employment of subtype-specific integrin-neutralizing antibodies or gene-silencing siRNAs has emerged as a valuable strategy for impairing HSV infectivity. Here, we shift the focus to a more affordable pharmaceutical approach based on small RGD-containing cyclic pentapeptides. Starting from our recently developed αvβ6-preferential peptide [RGD-Chg-E]-CONH_2_ (**1**), a small library of N-methylated derivatives (**2**–**6**) was indeed synthesized in the attempt to increase its affinity toward αvβ8. Among the novel compounds, [RGD-Chg-(*N*Me)E]-CONH_2_ (**6**) turned out to be a potent αvβ6/αvβ8 binder and a promising inhibitor of HSV entry through an integrin-dependent mechanism. Furthermore, the renewed selectivity profile of **6** was fully rationalized by a NMR/molecular modeling combined approach, providing novel valuable hints for the design of RGD integrin ligands with the desired specificity profile.

## Introduction

**Chart 1:**
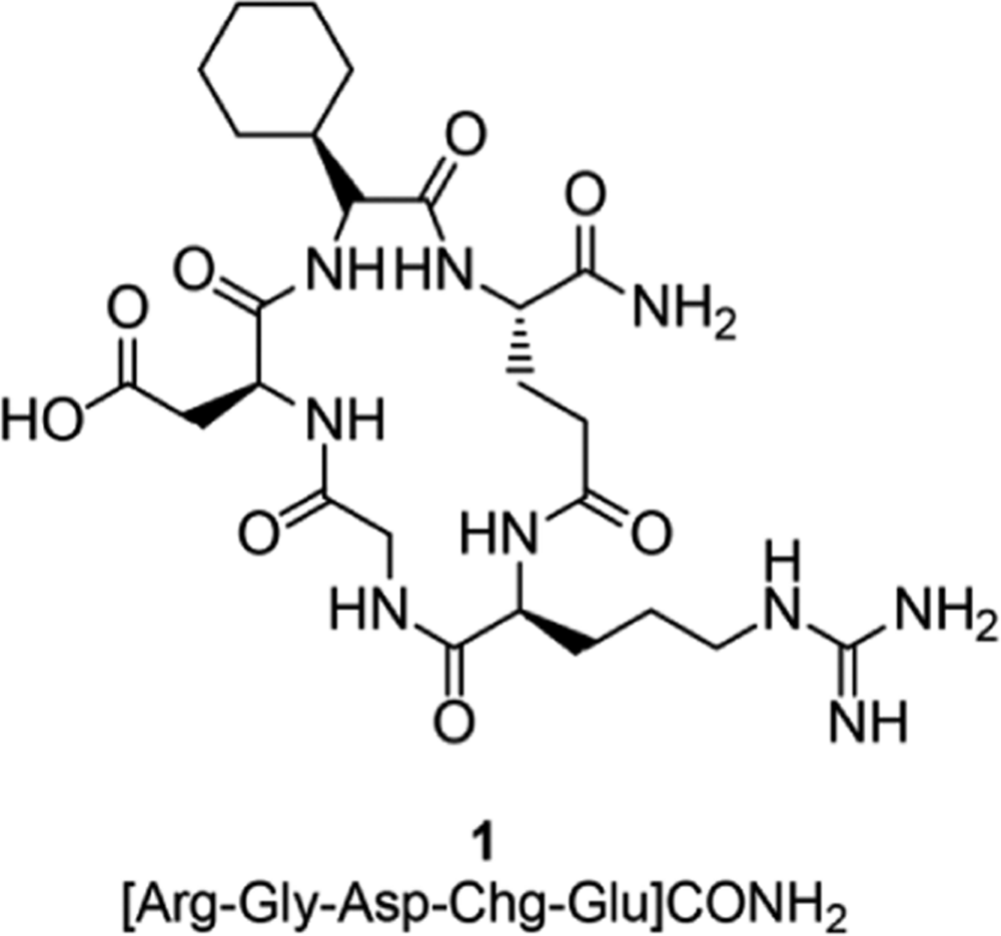
Chemical Structure of **1**

Integrins are well-known cell surface receptors, playing pivotal roles in both cell-to-cell and cell-to-extracellular matrix (ECM) cross talk. Indeed, these proteins span the plasma membrane to transmit bidirectional signals,^1^ thus modulating physiological functions, such as neoangiogenesis, cellular proliferation, migration, differentiation, endocytosis, and apoptosis.^2,3^ Therefore, integrin dysfunctions can trigger the onset of many diseases, such as chronic inflammation, cancer, and fibrosis.^3−8^ Integrins are a large family of 24 unique α–β heterodimers,^9−11^ which includes a subfamily of eight receptors (αvβ1, αvβ3, αvβ5, αvβ6, αvβ8, α5β1, αIIbβ3, and α8β1) that recognize the Arg-Gly-Asp (RGD) sequence in their physiological ligands.^12^ Because of their wide tissue distribution and involvement in various fundamental cellular functions, numerous pathogens, including viruses, likely evolved to exploit integrins for their infectious cycle.^13^ For instance, the Herpesviridae family can take advantage of integrins to broaden the spectrum of available host receptors needed for cellular attachment and entry phases.^14^ Within this family, we enumerate a number of heterogeneous pathogens, such as herpes simplex virus (HSV), varicella-zoster virus (VZV), human cytomegalovirus (HCMV), Epstein–Barr virus (EBV) or Kaposi’s sarcoma-associated herpesvirus (KSHV), and human herpesviruses 6 and 7 (HHV-6/-7). These can penetrate into different cells by both fusing the viral envelope with the plasma membrane and exploiting a synchronized endocytic uptake in neutral/acidic vesicles.^15,16^ Notably, not all herpesviruses use the same integrin receptor to enlarge their cellular tropism.^17^ In fact, while there is evidence that β1 subtypes are mainly involved in HCMV entry, αvβ6 and αvβ8 integrins serve as co-receptors for the foot-and-mouth disease virus (FMDV), EBV, and HSV cellular penetration.^14,18−21^ Specifically, the HSV cell entry–fusion is a multistep process mediated by three essential envelope glycoproteins: gD, the heterodimer gH/gL, and gB.^22^ The tropism of the virus is primarily determined by the recognition of gD by two cognate receptors on host cells, namely, nectin1 and herpesvirus entry mediator (HVEM). This event induces conformational changes in the gD ectodomain, which takes this protein into its functional state.^22−24^ Once activated, gD is able to recruit or stimulate the gH/gL heterodimer, thus triggering the switch of gB into its membrane-permeable fusogenic state and, ultimately, cellular fusion.^25^ In this context, recent studies have demonstrated that αvβ6 and αvβ8 integrins can alternatively interact with gH/gL, forcing the dissociation of gL from the parent heterodimer. This event can, in turn, favor the activation of gH and, eventually, gB.^26,27^ Remarkably, integrin-mediated activation of gH and gB can serve as a trigger checkpoint to synchronize the glycoprotein activation cascade with virion endocytosis. Accordingly, integrin-mediated regulation can ensure that the fusion machinery is not prematurely activated until endocytosis takes place.^26^ As a proof of concept, a consistent drop in HSV infectivity has been observed following the contemporary inhibition of αvβ6 and αvβ8 either by cell exposure to subtype-selective monoclonal antibodies (mAbs) or through siRNA transfection.^26^ This would suggest that the dual inhibition of these RGD integrins can be a promising strategy to develop brand-new anti-HSV therapeutic agents. Nonetheless, while many ligands are available for αIIbβ3, αvβ3, and α5β1,^28−37^ few selective binders are known for αvβ6 and αvβ8.^38−43^ In this regard, we recently identified a cyclic pentapeptide, namely, [RGD-Chg-E]-CONH_2_ (**1**) (Chart 1), as a potent and preferential αvβ6 ligand,^39^ which was successfully converted into an effective probe for molecular imaging.^42^ Considering that herpesviruses employ both αvβ6 and αvβ8 as co-receptors for cellular entry, a dual ligand of these integrins should represent a more efficient anti-HSV agent than the corresponding mono-αvβ6- or αvβ8-selective binders. Here, systematic N-methylation of the backbone amide bonds of **1** was performed since this strategy has frequently succeeded in enhancing the receptor binding affinity and tuning subtype specificity of cyclic peptides. Also, N-methylation can improve the peptides’ bioavailability or tolerance to enzymatic degradation.^44−49^ Hence, the newly synthesized peptides (**2**–**6**) were tested for their binding affinities on integrins of interest. Notably, [RGD-Chg-(*N*Me)E]-CONH_2_ (**6**) exhibited marked potency toward both αvβ6 and αvβ8 while sparing other closely related RGD-recognizing integrins. Cell biological assays were then performed to thoroughly evaluate the ability of **6** to impair the HSV-1 entry process through an integrin-dependent mechanism of action. Moreover, nuclear magnetic resonance (NMR) spectroscopy and molecular modeling studies were combined to elucidate the binding mode of **6** to αvβ6 and αvβ8 integrins, which can provide valuable hints for the future design of dual or subtype-specific RGD integrin-targeting agents.

## Results and Discussion

### Chemistry

**Scheme 1:**
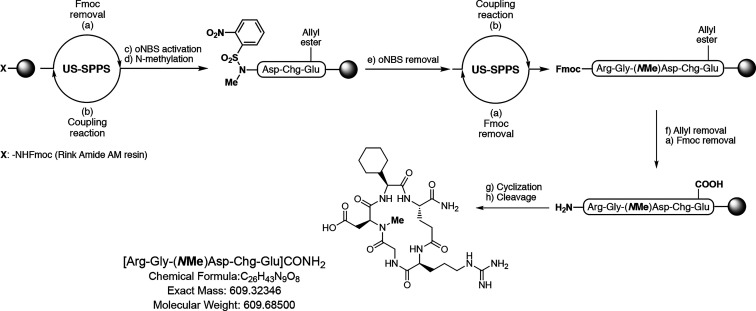
Synthesis of **4** as an Example of the General Procedure for Compounds **2**–**6** Conditions and reagents: (a) Piperidine 20% in *N*,*N*-dimethylformamide (DMF), 2 × 1 min, and US irradiation; (b) Fmoc-AA-OH, *O*-benzotriazole-*N*,*N*,*N*′,*N*′-tetra-methyluroniumhexafluorophosphate (HBTU), 1-hydroxybenzotriazole hydrate (HOBt), *N*,*N*-diisopropylethylamine (DIPEA), DMF, 5 min, and US irradiation; (c) *o*NBS chloride, triethylamine (TEA), dry dichloromethane (DCM), rt, 2 × 30 min; (d) Dimethylsulfate, DBU, dry NMP, room temperature, 2 × 30 min; (e) Mercaptoethanol, DBU, dry DMF, room temperature, 3 × 15 min; (f) Tetrakis(triphenylphosphine)palladium(0) (Pd(PPh_3_)_4_), dimethylbarbituric acid (DMBA), DCM/DMF 2:1, 2 × 60 min; (g) (1*H*-7-azabenzotriazol-1-yl-oxy)tris-pyrrolidinophosphonium hexafluorophosphate (PyAOP), 1-hydroxy-7-azabenzotriazole (HOAt), DIPEA, DMF, room temperature, 6 h; and h) Trifluoroacetic acid (TFA)/triisopropylsilane (TIS) 95:5, room temperature, 3 h.

Cyclic peptides **2**–**6** were assembled on the solid support according to a Fmoc/*t*-Bu approach and an ultrasound-assisted solid-phase peptide synthesis (US-SPPS) protocol previously reported by some of us (Scheme 1).^50^ Upon linear elongation, the methylation step was accomplished by activation of the α-NH_2_ group as *ortho*-nitrobenzensulfonylamide (*o*NBS-amide) and subsequent alkylation in the presence of dimethylsulfate (DMS) and 1,8-diazabicyclo[5.4.0]undec-7-ene (DBU) as the base in *N*-methyl-2-pyrrolidone (NMP).^51^ Treatment with mercaptoethanol and DBU as a scavenger mixture released the so obtained secondary amine and allowed for subsequent completion of the peptide synthesis (in Scheme 1, we describe the synthesis of **4** as an example of our synthetic strategy). This protocol was repeated for each peptide according to the position to be methylated. The cyclization step was then carried out on solid support in standard conditions, previously removing allyl and Fmoc protective groups from the glutamate side chain and the N-terminal of the sequence, respectively. Cleavage from the Rink amide AM resin in acidic conditions afforded the crude mixture that was purified using reverse-phase preparative high-performance liquid chromatography (HPLC).

### Binding Affinity Assays

The binding affinities of the newly synthesized compounds **2**–**6** and the stem peptide **1** toward αvβ6 and αvβ8 were evaluated through a competitive enzyme-linked immunosorbent assay (ELISA) using immobilized ECM protein and soluble integrin (Table 1).^47^ Compounds **1** and **6** were also tested on αvβ3 and α5β1 integrin receptors.

**Table 1:** Evaluation of the Binding Affinities of **2**–**6** Plus the Stem Peptide **1** for αvβ6 and αvβ8 Integrin Subtypes^a^

	IC_50_ (nM)				
	sequence	αvβ6	αvβ8	αvβ3	α5β1
**1**	[Arg-Gly-Asp-Chg-Glu]-CONH_2_	1.3 ± 0.1	174 ± 31	364 ± 96	105 ± 11
**2**	[(*N*Me)Arg-Gly-Asp-Chg-Glu]-CONH_2_	105 ± 8	2252 ± 89	n.d.	n.d.
**3**	[Arg-(*N*Me)Gly-Asp-Chg-Glu]-CONH_2_	211 ± 26	3319 ± 122	n.d.	n.d.
**4**	[Arg-Gly-(*N*Me)Asp-Chg-Glu]-CONH_2_	>5000	4687 ± 570	n.d.	n.d.
**5**	[Arg-Gly-Asp-(*N*Me)Chg-Glu]-CONH_2_	>5000	>5000	n.d.	n.d.
**6**	[Arg-Gly-Asp-Chg-(*N*Me)Glu]-CONH_2_	1.6 ± 0.1	60 ± 2	1199 ± 121	112 ± 26
cilengitide^b^		n.d.	n.d.	1.4 ± 0.1	22 ± 1
RTDLDSLRT^c^		38 ± 7	122 ± 38	n.d.	n.d

a Compounds **1** and **6** were also tested on αvβ3 and α5β1.

b Cilengitide was used as an internal reference compound in αvβ3 and α5β1 ELISA assays.

c RTDLDSLRT was used as an internal reference compound in αvβ6 and αvβ8 ELISA assays.

As regards the αvβ6 affinity, N-methylation of amino acids in the parent pentapeptide **1** turned out to be mostly detrimental for the binding to αvβ6, with the sole exception of the Glu^5^ to (*N*Me)Glu^5^ modification (**6**), which allowed for the obtained compound to maintain an half-maximal inhibitory concentration (IC_50_) value (1.6 nM) comparable to that of **1** (1.3 nM). Similarly, each N-methylation cycle resulted in a decrease of the ligand-binding affinity for αvβ8 except for **6**, which proved to be 3-fold more potent than **1** (60 *vs* 174 nM). Considering the increased αvβ8 affinity, the selectivity profile of **6** toward the structurally related integrins αvβ3 and α5β1 was also evaluated. Interestingly, **6** displays no significant binding affinity for αvβ3, whereas a residual binding for α5β1 comparable to that of the parent compound **1** (112 *vs* 105 nM), was detected. Thus, through N-methylation of Glu,^5^ we were able to transform the αvβ6-preferential peptide **1** in a novel αvβ6/αvβ8 dual ligand, albeit still with a binding preference for the former receptor.

### Nuclear Magnetic Resonance Experiments

**Figure 1:**
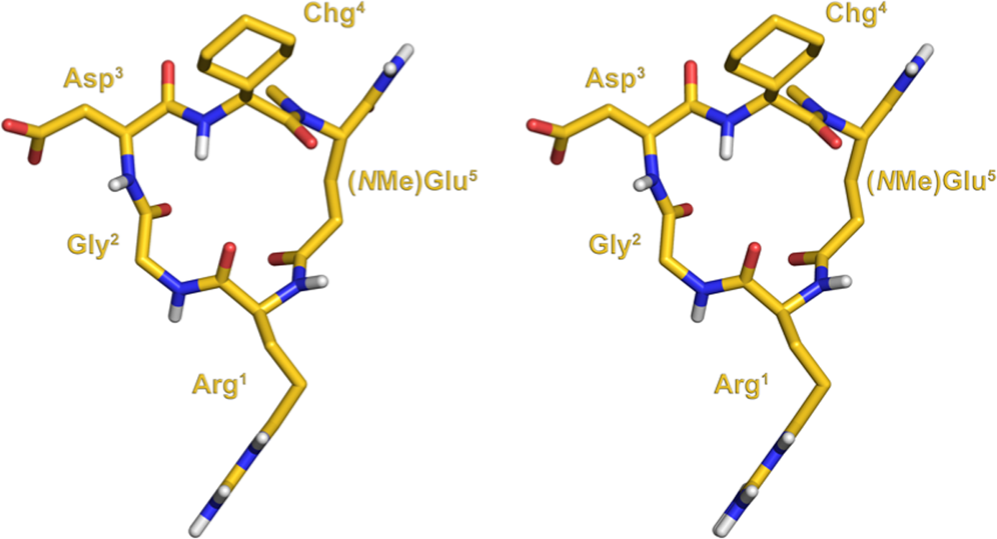
Stereoview of the NMR-derived conformation of **6**.

To identify the solution conformation of **6**, NMR experiments were performed in dimethyl sulfoxide (DMSO). A replica-averaged molecular dynamics (RAMD) protocol was applied to predict the tridimensional structure of **6**, using the nuclear Overhauser effect (NOE)-derived distances and the measured ^3^*J*_HN–Hα_ scalar couplings as experimental restraints. In RAMD, these are averaged over multiple parallel simulations of the system, leading to an accurate description of the underlying structural ensemble in accordance with the maximum entropy principle.^52−55^ The derived peptide conformation revealed (Figure 1) the presence of a βII′ turn-like motif centered around Gly^2^-Asp^3^ as suggested by the analysis of the dihedral angles ((φ^*i*+1^, ψ^*i*+1^, φ^*i*+2^, ψ^*i*+2^) = (63.4, −143.46, −83.98, −1.25)) of these residues. However, in contrast with the NMR conformation of stem peptide **1**, no stable intramolecular H-bond is formed between Arg^1^–CO and Chg^4^–NH, as shown by the analysis of the same interatomic distance averaged over the entire MD ensemble (Figure S1). This is in agreement with the lower-temperature coefficient calculated for Chg^4^–NH of **6** (−7.0 ppb/K, Table S2) compared to **1** (−0.3 ppb/K), indicating higher solvent accessibility of the amide group of this residue in the newly synthesized peptide. Also, a comparison of the tridimensional structures of **1** and **6** (Figure S2) highlights that the amide backbone N-methylation of Glu^5^ is responsible for changes in the dihedral space of the adjacent Chg^4^ residue, which vary from (φ, ψ) = (−89.8, 8.9) in **1** to (φ, ψ) = (−127.2, 80.8) in **6**. Interestingly, these differences result in a shift in the Chg^4^ side-chain orientation in the newly synthesized peptide.

### Molecular Modeling

**Figure 2:**
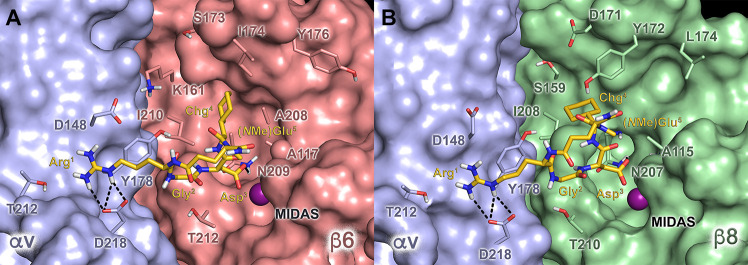
Docking poses of **6** (gold sticks) at the (A) αvβ6 (Protein Data Bank (PDB) code: 5FFO)^56^ and (B) αvβ8 (PDB code: 6OM2)^57^ integrins. The αv, β6, and β8 subunits are depicted as light blue, red, and green surfaces, respectively. The amino acid side chains important for ligand binding are represented as sticks. The metal ion at the MIDAS is represented as a purple sphere. Hydrogen bonds are shown as black dashed lines.

To investigate the molecular basis of the integrin activity and selectivity profile of **6**, docking calculations of the NMR-derived structure of this peptide were performed in the crystal structures of αvβ6^56^ and αvβ8.^57^ In fact, recent evidence suggests that, in the case of small RGD cyclopeptide ligands, the use of the solution NMR structure in docking calculations can improve their ability to reproduce the receptor-bound conformation.^58^ The top-ranked docking poses show that **6** can recognize these receptors by mimicking the canonical RGD interaction pattern (Figure 2). Specifically, the ligand Asp^3^ carboxylate group chelates the divalent cation at the metal-ion dependent adhesion site (MIDAS), forming an additional H-bond with the backbone of (β6)-N209 and (β8)-N207 in αvβ6 and αvβ8, respectively (Figure 2); on the other hand, the Arg^1^ guanidinium moiety of **6** establishes a side-on tight salt bridge with the conserved (αv)-D218 residue. Beside the RGD motif, the Chg^4^ side chain is hosted in the cavity defined by the specificity-determining loop (SDL), forming multiple lipophilic contacts with the side chains of (β6)-A117, (β6)-L174, (β6)-Y176, (β6)-A208, and (β6)-I210 in αvβ6 and (β8)-A115, (β8)-Y172, (β8)-L174, and (β8)-I208 in αvβ8. Indeed, these interaction schemes are consistent with the low–mid-nanomolar IC_50_ values exhibited by **6** toward αvβ6 and αvβ8. Then, we investigated the reasons for the improved αvβ8 affinity of this compound with respect to its parent peptide **1**. To this aim, an accurate comparison between the RGD binding sites of the two receptors was performed, showing an increased steric hindrance in the SDL cavity of αvβ8 mainly due to the replacement of (β6)-I174 with (β8)-Y172. Indeed, this residue might clash with the cyclohexyl ring of **1**, not allowing this peptide to properly accommodate in the β8 subunit, as suggested by the superimposition of the **1**/αvβ6 docking complex with the αvβ8 X-ray structure (Figure S3). Notably, this clash is not observed in the **6**/αvβ8 complex due to the different orientations assumed by the Chg^4^ side chain in the N-methylated compound, as revealed by NMR analysis. Thus, we can conclude that the changes in the peptide conformation induced by Glu5 N-methylation, together with single point mutations in the SDL cavity of the β6 and β8 subunits, are responsible for the different selectivity profile of compounds **1** and **6**. This, in turn, further proves how minimal chemical modifications in small cyclic peptides can account for large differences in their binding affinities.

### Biological Evaluation

**Figure 3:**
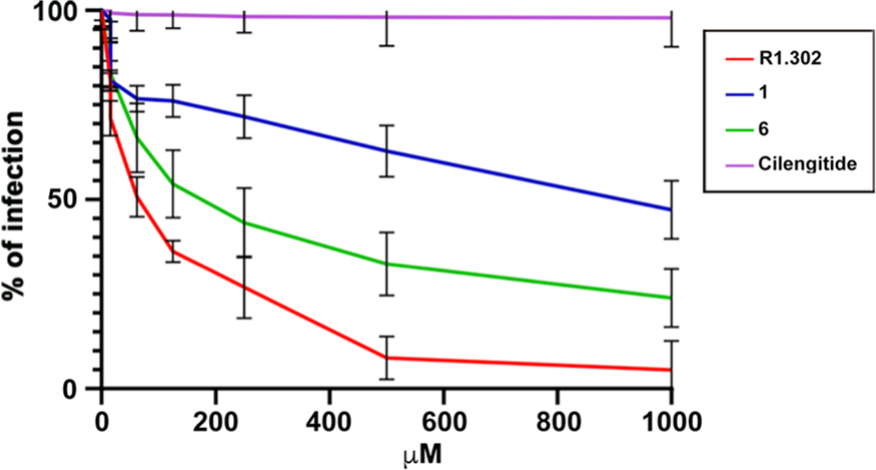
Inhibition of HSV-1 infection. 293T cells were exposed to increasing concentrations of the indicated peptides for 1 h prior to infection and during virus attachment for another 90 min. Infection was induced using the recombinant R8102 HSV-1 strain and measured after 8 h as β-galactosidase activity, using *o*-nitrophenyl-d-galactopyranoside (ONPG) as a substrate. The assays were run in triplicate. Bars show standard deviation (SD).

The binding of HSV-1 gH/gL to either αvβ6 or αvβ8 results in the dissociation of gL from the heterodimer, which, in turn, can favor HSV entry into cells.^27^ Here, cell experiments were performed to evaluate the ability of the newly synthesized dual αvβ6/αvβ8 ligand **6** to impair the HSV-1 infectivity in comparison with both αvβ6-preferential binder **1** and the well-characterized αvβ3/αvβ5 integrin ligand cilengitide. R1.302, a nectin1-neutralizing mAb, was used as a positive control. In the first set of experiments, 293T cells, expressing both the αvβ6 and αvβ8 integrins, were alternatively exposed to increasing concentrations of the integrin-binding peptides and R1.302 before and during the infection by the recombinant R8102 HSV-1 strain. This virion carries, indeed, a lacZ reporter gene under the control of the α27 promoter, whose expression analysis allows for readily quantifying the infectivity.^59^ As shown in Figure 3, both **1** and **6** were able to inhibit HSV-1 infection in a dose-dependent manner. Nonetheless, the efficacy of **6** ranged from 70 to 80% at 500–1000 μM peptide concentration, while the parent compound **1** turned out to be less effective, showing a 50% maximum inhibition at 1000 μM. As proof of the αvβ6/αvβ8-related inhibitory activities of **1** and **6**, no alteration in the HSV entry process was detected when cells were treated with cilengitide. Remarkably, these preliminary results were in agreement with the known interchangeable and additive roles played by αvβ6 and αvβ8 integrins upon HSV-1 infection.^26^

**Figure 4:**
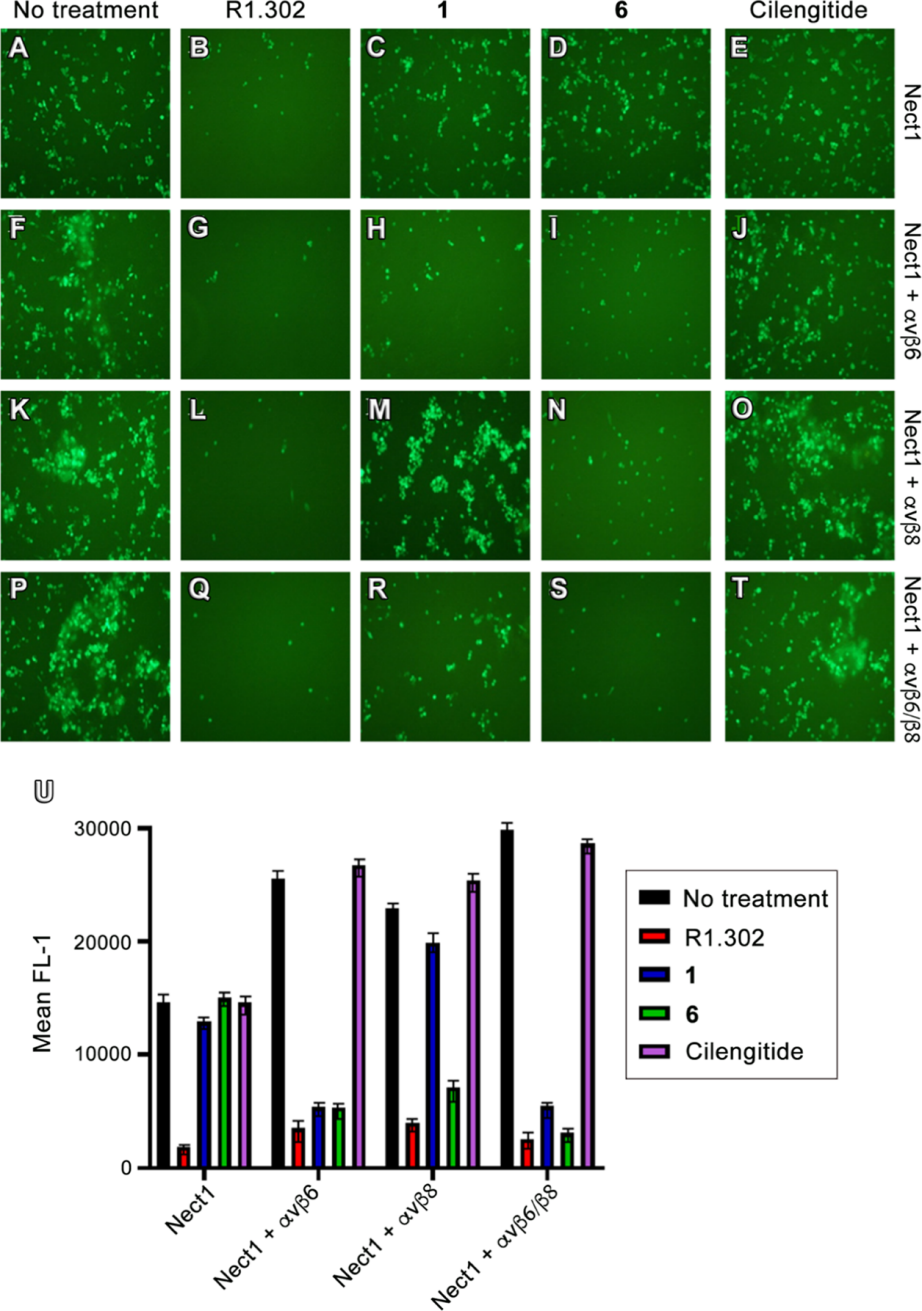
Inhibition of HSV-1 infection by peptides. J cells were transfected with a low amount (75 ng of DNA/24 well) of nectin1 alone (A–E), or with the same amount of nectin1 plus αvβ6 integrin (300 ng DNA/24 well) (F–J), or plus αvβ8 integrin (300 ng DNA/24 well) (K–O), or plus both the integrin receptors (P–T). Then, 48 h after transfection, the cells were exposed to 700 μM peptides (**1**, **6**, and cilengitide) for 1 h prior to infection and 90 min during virus attachment. The cells were infected with K26GFP. The nonpenetrated virus was inactivated by an acid wash. Infectivity was measured at 16 h after infection as EGFP expression. (A)–(T) panels show the EGFP expression in each sample for a typical experiment. (U) K26GFP infection was quantified as EGFP protein expression in the flow cytometry assay as the mean fluorescence intensity (MFI) of gated cells. Histograms represent the average of triplicates ± SD.

However, due to the contemporary expression of αvβ6 and αvβ8 in 293T cells, further experiments were required to confirm the hypothesis that **1** and **6** exerted different effects on HSV infection, either by preferentially binding to αvβ6 (**1**) or by simultaneously targeting αvβ6 and αvβ8 (**6**). To this aim, we selected the J cells, which are negative for gD receptors and therefore cannot be infected by HSV-1 unless gD cognate receptors (i.e., nectin1) are transgenically expressed. Also, J cells present endogenous hamster integrins, likely at low levels, and hence they can be engineered with various human integrin subtypes to evaluate the role of these receptors in HSV-1 infection. Thus, variously transfected J cells were treated with each compound at 700 μM, corresponding to the concentration required by the less active compound (**1**) to inhibit by 40% the HSV infection in 293T cells. In particular, J cells were transfected with low amounts of nectin1 plasmid, plus either αvβ6 or αvβ8 integrin plasmid or both, and then infected with the recombinant K26GFP HSV-1 strain. Indeed, the capsid protein ICP26 of this strain is fused to the green fluorescent protein (GFP),^60^ whose cellular expression can be used to measure the viral infection. Thus, the K26GFP entry in J cells after compound treatment was evaluated using fluorescent microscopy (Figure 4A–T), and the enhanced green fluorescent protein (EGFP) expression was quantified using flow cytometry as the mean fluorescence intensity (MFI) of gated cells (Figure 4U). First, the integrin specificities of **1** and **6** were confirmed in J cells expressing nectin1 alone (Figure 4A–E), where, indeed, only treatment with R1.302 (B) was effective in preventing HSV-1 infection. Then, we observed that in J cells expressing nectin1 plus both αvβ6 and αvβ8 integrins (Figure 4P–T), the HSV infection was significantly reduced after treatment with either R1.302 (Q) or compound **6** (S), while it was only partially impaired after the addition of **1** (R). In parallel, we also evaluated the HSV infection in J cells expressing nectin1 plus either αvβ6 or αvβ8 alone. Interestingly, when only the αvβ6 plasmid was transfected (Figure 4F–J), the virus entry was affected by both **1** and **6** (H and I); conversely, in J cells exclusively expressing nectin1 plus αvβ8 (Figure 4K–O), inhibitory effects were detected only in the presence of **6** (N). We also remark that in all of the examined samples, the R1.302 mAb blocked HSV entry, whereas no effect was exerted by cilengitide. Altogether, these outcomes prove the capability of peptides **1** and **6** to impair HSV-1 cellular penetration by blocking the interaction of viral gH with the αvβ6 and αvβ8 integrins and at the same time highlight the advantage of contemporary inhibition of these receptors, as demonstrated by the higher antiviral efficacy of the αvβ6/αvβ8 integrin dual ligand **6**.

Remarkably, while gH interacts with αvβ6 through its RGD domain, directly competing with our peptides for integrin binding, αvβ8 does not contact gH by recognizing its RGD triad. Therefore, another mechanism might be responsible for the activity of **6** on J cells expressing αvβ8 integrin alone as well as the higher activity of **6** than that of **1** in cells that express both αvβ6 and αvβ8.^38^ Thus, we hypothesized that **6** can prevent HSV-1 entry into J cells expressing nectin1 and the αvβ8 integrin alone by inducing internalization of the integrin itself. To validate our hypothesis, J cells were transfected with nectin1 and the two integrins αvβ6 and αvβ8 in the above-described manner. Then, 48 h after transfection, the cells were incubated for 60 min at 37 °C with **1** or **6**. The surface expression of integrin β6 and β8, in the presence or the absence of peptides, was then evaluated by flow cytometry using FAB4155A (mAbβ6) and FAB4775A (mAbβ8) monoclonal antibodies that recognize other integrin regions than the RGD-binding domain.

**Figure 5:**
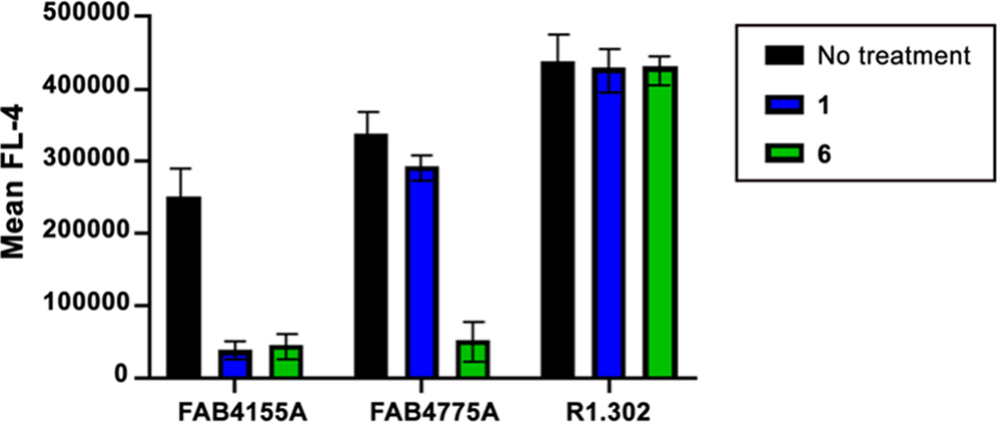
Integrin internalization assay. J cells were transfected with a low amount (75 ng of DNA/24 well) of nectin1 plus αvβ6 integrin (300 ng of DNA/24 well) and αvβ8 integrin (300 ng of DNA/24 well). Then, 48 h after transfection, the cells were exposed to 700 μM peptides for 1 h at 37 °C. Cells derived from samples treated or not treated with peptides were incubated for 1 h at 4 °C with FAB4155A (mAbβ6), FAB4775A (mAbβ8), and R1.302 (mAbnectin1) mAbs. Samples incubated with R1.302 were washed and subsequently incubated for 1 h at 4 °C with the allophycocyanin (APC) mouse secondary antibody. Integrin and nectin1 surface expression levels were quantified as flow cytometry expression of the APC mean fluorescence intensity (MFI) of gated cells. Histograms represent the average of triplicates ± SD.

As shown in Figure 5, the membrane expression of integrin β6 is reduced following treatment with either **1** or **6** consistent with the results shown in Figures 3 and 4, while the integrin β8 level is reduced following treatment with **6** but not with **1**. On the other hand, the surface expression of nectin1 is not altered following treatment with either peptide. These results clearly indicate that both **1** and **6**, once bound to the RGD-binding domain of the targeted integrin, determine receptor internalization. This results in a lower expression of the two integrins at the cell surface and, accordingly, in a reduced probability to be used as receptors or co-receptors.

**Figure 6:**
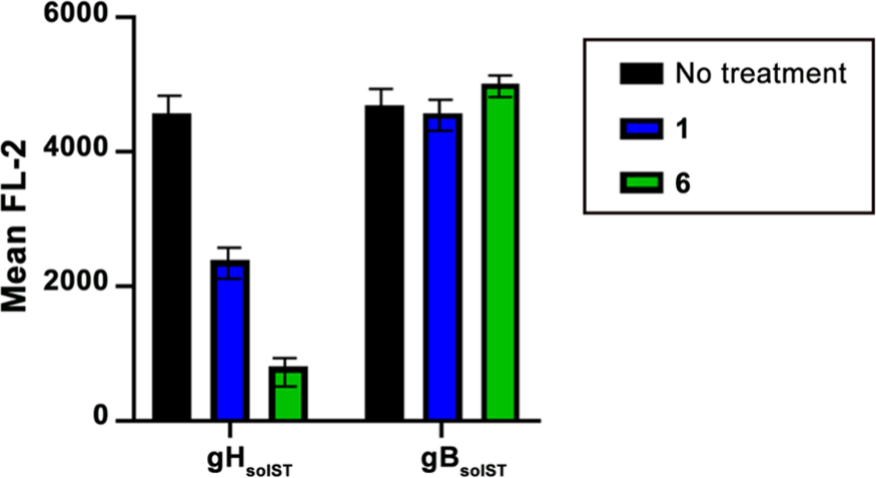
gH binding assay. J cells were transfected with a low amount (75 ng of DNA/24 well) of nectin1 plus αvβ6 integrin (300 ng of DNA/24 well) and αvβ8 integrin (300 ng of DNA/24 well). Then, 48 h after transfection, the cells were exposed to 700 μM peptides for 1 h at 37 °C. Cells derived from samples treated or not treated with peptides were incubated for 1 h at 4 °C with gH_solST_ or gB_solST_. Samples were washed and subsequently incubated for 1 h at 4 °C with phycoerythrin (PE)-conjugated mAb to the One-STrEP tag (Strep-Tactin). gH_solST_ and gB_solST_ binding to the cell surface were quantified as the flow cytometry expression of the PE mean fluorescence intensity (MFI) of gated cells. Histograms represent the average of triplicates ± SD.

To further verify this hypothesis, the soluble form of gH (gH_solST_) was used in a soluble protein in the cell-binding assay on J cells transfected with nectin1 and the αvβ6 and αvβ8 integrins, treated or untreated with each peptide. A soluble form of gB (gB_solST_) was used as a control for the specificity of compounds **1** and **6** in the experiment, since this HSV envelope glycoprotein does not bind any integrins while it recognizes the heparan sulfate present on the cell membrane. Figure 6 shows that the binding of gH_solST_, but not of gB_solST_, is strongly reduced in cells treated with **6** and only partially reduced in cells treated with **1**, indicating that integrin internalization induced by our peptides determines a weak capacity of binding gH, which, in turn, attenuates HSV-1 infection.

## Conclusions

Herpesviruses have evolved to utilize integrin cell biology and physiology to promote their persistence and widespread dissemination in nature. Particularly, the αvβ6 and αvβ8 RGD integrins have been found to act as key co-receptors for entry proteins of some viruses including HSV.^26,27^ Indeed, both the receptors can interchangeably interact with the HSV-1 gH/gL surface glycoproteins to promote viral penetration into the host cell. This evidence has prompted researchers to investigate αvβ6 and αvβ8 as novel targets to block HSV infection; however, only the use of subtype-specific integrin-neutralizing antibodies or gene-silencing strategies has been explored so far.^26,27^ Here, we demonstrate that HSV infection can be impaired through a more affordable pharmaceutical approach based on the use of small, αvβ6/αvβ8 dual, RGD-containing cyclic pentapeptides. To this end, we generated a small library of N-methylated derivatives of the αvβ6 specific ligand [RGD-Chg-E]-CONH_2_ (**1**) recently discovered by us^39^ and evaluated their affinity and selectivity profile on a selected RGD integrin panel. Among the newly synthesized compounds, [RGD-Chg-(*N*Me)E]-CONH_2_ (**6**) displayed an increased αvβ8 affinity compared to the parent ligand, representing one of the most potent αvβ6/αvβ8 dual ligands discovered so far. The molecular basis of the increased αvβ8 potency of **6** with respect to the stem peptide **1** was rationalized with the aid of NMR experiments and computational studies. Furthermore, **1** and **6** underwent an extensive biological evaluation on different cell lines, which demonstrated the ability of both peptides to impair the HSV infection. Nonetheless, **6** showed remarkably higher efficacy than **1** in inhibiting HSV cellular penetration, highlighting the importance of simultaneously targeting both αvβ6 and αvβ8 to increase the antiviral activity. Moreover, we further demonstrated that our peptides can inhibit HSV cellular entry by inducing receptor internalization rather than competing with the viral glycoproteins for binding to the canonical RGD site.

To date, drug therapy to counteract HSV-1 infection is based exclusively on the use of aciclovir (or its prodrugs), which acts by blocking viral replication. This treatment, albeit effective, does not hinder the entry of the pathogen into the host and therefore carries frequent side effects, such as periodic virus reactivation events and the insurgence of resistance phenomena.^61^ In this context, the adjuvant use of small peptides as inhibitors of the HSV-1 entry might compensate for some of the drawbacks of current therapeutic regimens. Furthermore, small chemical entities can take advantage of better tissue penetration, lower production cost, and lower immunogenicity than neutralizing antibodies directed on the same proteins.^62^ Altogether, our outcomes encourage the development of αvβ6/αvβ8 dual compounds as a new potential therapeutic approach to the HSV disease and, at the same time, open up the possibility of employing small ligands of RGD integrins as weapons against a wide range of viruses that employ this class of receptors as gateways to invade host cells.

## Experimental Section

### Materials

Fmoc-Rink amide-AM resin, *O*-benzotriazole-*N*,*N*,*N*′,*N*′-tetra-methyluroniumhexafluorophosphate (HBTU) (purity 99%), *N*,*N*-diisopropylethylamine (DIPEA) (purity 99%), trifluoroacetic acid (TFA) (purity 99%), piperidine, Fmoc-l-Arg(Pbf)-OH, Fmoc-Gly-OH, Fmoc-l-Asp(O*t*Bu)-OH, Fmoc-l-Glu(OAll)-OH, and Fmoc-l-Chg-OH were purchased from IRIS Biotech (Marktredwitz, Germany).

*ortho*-Nitrobenzenesulfonyl chloride (*o*NBS-Cl) (purity 97%), triethylamine (TEA), dimethylsulfate (DMS) (purity 99%), 1,8-diazabicyclo[5.4.0]undec-7-ene (DBU) (purity 98%), 2-mercaptoethanol (purity 99%), triisopropylsilane (TIS) (purity 98%), 1-hydroxybenzotriazole hydrate (HOBt) (purity >97% dry weight, water ≈12%), (1*H*-7-azabenzotriazol-1-yl-oxy)tris-pyrrolidinophosphonium hexafluorophosphate (PyAOP) (purity 96%), 1-hydroxy-7-azabenzotriazole (HOAt, purity 96%), tetrakis(triphenylphosphine)palladium(0) or Pd(PPh_3_)_4_ (purity 99%), dimethylbarbituric acid (DMBA) (purity 99%, water content <6%), Tween 20, phosphate-buffered saline (PBS) tablets, OmniPur TRIS hydrochloride, bovine serum albumin (BSA), 3,3′,5,5′-tetramethylbenzidine (TMB) liquid substrate system, fibronectin human plasma (0.1% solution) (Sigma-Aldrich code F0895), cilengitide (Sigma-Aldrich code SML1594), antimouse immunoglobulin G (IgG)–peroxidase antibody produced in rabbit (Sigma-Aldrich code A9044), anhydrous *N*,*N*-dimethylformamide (DMF), *N*-methylpirrolidone (NMP), dichloromethane (DCM), and dimethyl sulfoxide (DMSO) were purchased from Sigma-Aldrich (Milano, Italy).

Peptide synthesis solvents and acetonitrile for HPLC were of reagent grade, acquired from commercial sources (Sigma-Aldrich, Milano, Italy), and used without any further purification unless otherwise stated. Water for HPLC was purchased from Levanchimica s.r.l. (Bari, Italy). Peptides were purified by preparative HPLC (Shimadzu HPLC system) equipped with a C18-bounded preparative reversed-phase HPLC (RP-HPLC) column (Phenomenex Kinetex 21.2 mm × 150 mm, 5 μm). Peptide purity was determined by analytical HPLC (Shimadzu Prominence HPLC system) equipped with a C18-bounded analytical RP-HPLC column (Phenomenex Kinetex, 4.6 mm × 150 mm, 5 μM) using gradient elution (10–90% acetonitrile in water (0.1% TFA) for over 20 min; flow rate = 1.0 mL/min; a diode array UV detector). Molecular weights of compounds were confirmed by electrospray ionization (ESI) high-resolution mass spectrometry (HRMS) using a Q Exactive Orbitrap LC-MS/MS mass spectrometer (Thermo Fisher Scientific, Waltham, MA).

Human vitronectin (supplier code CC080) and mouse anti-integrin αv antibody (supplier code mAb1978) were purchased from Merck-Millipore KGaA (Darmstadt, Germany).

Flat-bottom 96-well ELISA plates were purchased from BRAND (Wertheim, Germany).

Human αvβ3-integrin (supplier code 3050-AV), human α5β1 integrin (supplier code 3230-A5), human αvβ6 integrin (supplier code 3817-AV), human αvβ8 integrin (supplier code 4135-AV), and recombinant human latency-associated peptide (LAP) (transforming growth factor (TGF)-β1) (supplier code 246-LP) were purchased from R&D Systems (Biotechne brand) (MN).

Purified mouse antihuman CD51/61 (supplier code 555504) and purified mouse antihuman CD49e (supplier code 555617) were purchased from BD Biosciences (CA).

### Synthetic Procedure

A Rink Amide AM-PS resin (136 mg, 0.75 mmol, 0.55 mmol/g) was swelled in a mixture of DCM/DMF, 1:1, for 20 min and then drained on solid-phase peptide manifold (Macherey-Nagel Chromab.) without any further treatment. Linear oligomers were assembled according to a Fmoc/*t*-Bu synthetic approach and an ultrasound-assisted solid-phase peptide synthesis (US-SPPS) protocol recently reported by some of us.^50^ In general, Fmoc deprotections were carried out treating the solid phase with a 20% piperidine solution in DMF under ultrasound irradiation in a SONOREX RK 52 H cleaning bath (2 × 1 min, approx. 1.5 mL each treatment). Coupling reactions were accomplished using 3 equiv of amino acids preactivated with an equimolar amount of coupling reagents (HBTU and HOBt) and 6 equiv of DIPEA as the base (with respect to the resin functionalization). In detail, the building blocks and activating reagents were dissolved in DMF (2 mL) before DIPEA was added; the mixture was then added to the resin in a SPPS reactor, which was irradiated with ultrasound for 5 min. Before and after each synthetic step, the resin was washed with DMF (three times) and DCM (three times) to rinse out the unreacted materials. Completion of coupling reactions was determined by the Kaiser ninhydrin test or the trinitrobenzenesulfonic acid (TNBS or picrylsulfonic acid) test.

The N-methylation step was carried out through *ortho*-nitrobenzenesulfonylamide activation of the desired α primary amine. First, the resin was treated twice with a previously prepared solution containing 5 equiv (83 mg) of *o*NBS-Cl and 10 equiv of TEA (105 μL) in anhydrous DCM (2 mL). The resulting solution was added to the resin, which was allowed to be shaken for 30 min during each cycle. After each treatment, the resin was drained and washed with DMF (three times) and DCM (three times). Completion of the protection/activation step was monitored by the Kaiser ninhydrin test. The so obtained nitrobenzenesulfonylamide was methylated by treating the resin twice with a solution containing 10 equiv (71 μL) of DMS and 3 equiv (34 μL) of DBU in anhydrous NMP (2 mL). The mixture was added to the resin, which was shaken for 30 min during each cycle. After each treatment, the resin was drained and washed with DMF (three times) and DCM (three times). To release the secondary amine, the *o*NBS protecting group was removed by treating the resin three times with a solution containing 10 equiv (53 μL) of 2-mercaptoethanol and 5 equiv (56 μL) of DBU in anhydrous DMF. The mixture was added to the resin, which was allowed to be shaken for 15 min during each cycle. After each treatment, the resin was drained and washed with DMF (three times) and DCM (three times), and at the end of the three treatments, the release of the secondary amine was ascertained by the on-resin chloranil test. The assembly of monomers on N-methylated amino acids was performed under the same conditions as stated above but by repeating the coupling reaction twice before proceeding with the subsequent synthetic steps.

Once on-resin linear elongation was achieved, the allyl ester protective group removal (on the first amino acid side chain) was achieved by treating the solid support with a solution of 0.1 equiv of Pd(PPh_3_)_4_ (9 mg) and 8 equiv of DMBA (94 mg) in anhydrous DCM/DMF (2:1, 2 mL). The mixture was gently shaken under an argon atmosphere for 1 h and the treatment was repeated one more time. The resin was drained, washed with DMF (three times) and DCM (three times), and then treated with a solution of 0.06 M potassium *N*,*N*-diethyldithiocarbamate in DMF (25 mg in 2 mL of solvent) for 1 h to wash away catalyst traces. Before performing on-resin cyclization, the N-terminal free primary amine was released from the last amino acid by US-SPPS Fmoc-deprotection as previously described. Then, head-to-side-chain cyclization was carried out using 3 equiv of PyAOP and HOAt as dehydrating agents and 6 equiv of DIPEA as the base in DMF (3 mL). The coupling reagents were dissolved in the solvent prior to the base being added, and then the yellowish solution was added to the resin, which was allowed to be shaken for 6 h. Fulfillment of cyclization was ascertained by the Kaiser ninhydrin test.

The so obtained resin-bound peptides were washed with DMF (three times), DCM (three times), and Et_2_O (three times) and then dried exhaustively. Then, the amino acid side chain-protecting groups were removed one-pot upon peptide cleavage from the solid support by treatment with a solution of TFA/TIS (95:5, 2 mL) and then gently stirred for 3 h at room temperature. The resin was filtered, and the crude peptides were precipitated from the cleavage solution diluting to 15 mL with cold Et_2_O and then centrifuged (6000 rpm × 15 min). The supernatant was carefully removed, and the crude precipitate was resuspended in 15 mL of Et_2_O and then centrifuged as previously described. After removing the supernatant, the resulting wet solid was dried for 1 h under reduced pressure, dissolved in water/acetonitrile (95:5), and purified by reverse-phase HPLC (eluent A: water + 0.1% TFA; eluent B: acetonitrile + 0.1% TFA; with a linear gradient from 10 to 70% of eluent B for over 20 min, flow rate: 10 mL/min). Fractions of interest were collected and evaporated from organic solvents, frozen, and then lyophilized. The obtained products were characterized by analytical HPLC (Figures S4–S8) (eluent A: water + 0.1% TFA; eluent B: acetonitrile + 0.1% TFA; from 10 to 90% of eluent B for over 20 min, flow rate: 1 mL/min), and the identity of peptides was confirmed by high-resolution mass spectra (Figures S9–S13) (Q Exactive Orbitrap LC-MS/MS, Thermo Fisher Scientific). All of the final products were obtained with a purity ≥95%.

### Integrin-Binding Assay

The affinity and selectivity of the integrin peptide ligands were ascertained according to a modified method previously reported by some of us.^33,47^ The binding was determined in a competitive solid-phase binding assay using extracellular matrix proteins, soluble integrins, and pertinent integrin-specific antibodies in an enzyme-linked immunosorbent assay (ELISA).

#### LAP(TGF-β1)-αvβ6 Assay

A flat-bottom 96-well ELISA plate was coated overnight at 4 °C with 100 μL/well of 0.4 μg/mL LAP previously diluted in carbonate buffer (15 mM Na_2_CO_3_, 35 mM NaHCO_3_, pH 9.6). Afterward, each well was then washed three times with 200 μL of PBS-T buffer (137 mM NaCl, 2.7 mM KCl, 10 mM Na_2_HPO_4_, 2 mM KH_2_PO_4_, 0.01% Tween 20, pH 7.4) and blocked for 1 h at room temperature with 150 μL/well TSB buffer (20 mM Tris–HCl, 150 mM NaCl, 1 mM CaCl_2_, 1 mM MgCl_2_, 1 mM MnCl_2_, pH 7.5, 1% BSA). Then, each well was washed three times with 200 μL of PBS-T.

Equal volumes (50 μL) of the internal standard (or test compounds) were mixed with 0.5 μg/mL human integrin αvβ6 (50 μL) giving a final dilution series in TSB buffer of 0.00064–10 μM for the inhibitors and 0.25 μg/mL for integrin αvβ6. These solutions (100 μL/well) were added to the flat-bottom ELISA plate and incubated for 1 h at room temperature. The solutions were discarded from the wells, and the plate was washed three times with PBS-T buffer (3 × 200 μL). At this point, 100 μL/well of 1:500 diluted mouse antihuman αv integrin was added to the plate and incubated for 1 h at room temperature and the plate was washed three times with PBS-T buffer (3 × 200 μL), and 100 μL/well of 2.0 μg/mL secondary peroxidase-labeled antibody (antimouse IgG-POD) was added to the plate and incubated for 1 h at room temperature. The solutions were discarded from the wells, and the plate was washed three times with PBS-T (3 × 200 μL). The binding assay was developed by adding 50 μL/well of the 3,3′,5,5′-tetramethylbenzidine (TMB) liquid substrate system and incubating for 1 min at room temperature. The reaction was stopped by adding 50 μL/well of 3 M H_2_SO_4_. The peroxidase product formation was detected by measuring the absorbance at 405 nm using a plate reader (Tecan SpectraFLUOR plus, Männedorf, Switzerland). Each compound concentration was tested in duplicate, and the resulting inhibition curves were analyzed using OriginPro 7.5G software; the inflection point describes the IC_50_ value. Each plate contained a linear peptide (RTDLDSLRT) as the internal standard.^63^

#### LAP(TGF-β1)-αvβ8 Assay

The experimental procedure is as described for the αvβ6 assay, except for the following modifications. The plates were coated, washed, and blocked as described above. Soluble integrin αvβ8 was mixed with an equal volume of serially diluted inhibitors resulting in a final integrin concentration of 0.25 μg/mL and 0.0032–10 μM for the inhibitors. The primary and secondary antibodies are the same as in the integrin αvβ6 assay. Visualization and analysis were performed as described above. Each plate contained a linear peptide (RTDLDSLRT) as the internal standard.^3^

#### Vitronectin-αvβ3 Assay

The experimental procedure was as described for the αvβ6 assay, except for the following modifications. The plates were coated with 100 μL/well of 1.0 μg/mL vitronectin in carbonate buffer, washed, and blocked as described above. Soluble integrin αvβ3 was mixed with an equal volume of serially diluted inhibitors resulting in a final integrin concentration of 1.0 μg/mL and 0.128–400 nM for cilengitide and 0.0032–10 μM for the inhibitors. As a primary antibody, 100 μL/well of 2.0 μg/mL primary antibody (mouse antihuman CD51/61) was used. The secondary antibody used was the same as that used for the integrin αvβ6 assay. Visualization and analysis were performed as described above. Each plate contained cilengitide as the internal standard.^44^

#### Fibronectin-α5β1 Assay

The experimental procedure was as described for the αvβ6 assay, except for the following modifications. The plates were coated with 100 μL/well of 0.5 μg/mL fibronectin in carbonate buffer, washed, and blocked as described above. Soluble integrin α5β1 was mixed with an equal volume of serially diluted inhibitors resulting in a final integrin concentration of 1.0 μg/mL and 0.00064–2 μM for cilengitide and 0.0032–10 μM for the inhibitors. As a primary antibody, 100 μL/well of 1.0 μg/mL primary antibody (mouse antihuman CD49e) was used. The secondary antibody used was the same as that used in the integrin αvβ6 assay. Visualization and analysis were performed as described above. Each plate contained cilengitide as the internal standard.^44^

### Nuclear Magnetic Resonance

First, **6** was dissolved in *d*_6_-DMSO. All NMR experiments were recorded at 298 K on a 600 MHz spectrometer (Bruker Avance600 Ultra Shield Plus) equipped with a triple-resonance TCI cryoprobe with a *z*-shielded pulsed-field gradient coil.

#### NMR Assignment

The following monodimensional (1D) and bidimensional (2D) homonuclear experiments were acquired for proton assignment: 1D ^1^H, 2D ^1^H–^1^H total correlation spectroscopy (TOCSY, *t*_mix_ = 60 ms), 2D ^1^H–^1^H rotating-frame overhauser effect spectroscopy (ROESY, *t*_mix_= 300 ms, spin-lock at 2.8 kHz), and 2D ^1^H–^1^H nuclear Overhauser effect spectroscopy (NOESY, *t*_mix_ = 200 ms). Carbon resonances were assigned from 2D ^1^H–^13^C heteronuclear single quantum coherence (HSQC). Spectra were processed using Topspin3.6 (Bruker) (Figures S14–S18). For the ^1^H–^1^H 2D experiments, free induction decays were acquired (32–64 scans) over 8000 Hz into 2k data blocks for 512 incremental values for the evolution time. The ^13^C HSQC experiment was performed using a spectral width of 8000 Hz (*t*_1_) and 11300 Hz (*t*_2_) and 2048 (160) data points (48 scans). Data were typically apodized with a square cosine window function and zero-filled to a matrix of size 2048 ×1024 (2048 × 256 for ^13^C HSQC) before Fourier transformation and baseline correction. Spectra were analyzed with CCPNMR2.4 software.^64^ Chemical shift assignments refer to sodium 2,2-dimethyl-2-silapentane-5-sulfonate (DSS). ^3^*J* coupling constants were directly obtained from well-digitized monodimensional spectra (40k points), analyzing well-resolved amides and Hα proton resonances. To estimate the solvent shielding or hydrogen bond strength of NH protons, the temperature dependency of NH chemical shifts was monitored acquiring ^1^H-1D spectra between 285 and 305 K in steps of 5 K increments.

#### Proton–Proton Internuclear Distances for Structure Calculation

NOE cross peak volumes were converted to ^1^H–^1^H internuclear distances by the linear approximation method using as reference the geminal proton distance of (*N*Me)Glu5 Hβa–Hβb (fixed at 1.75 Å). The calculated distances were then relaxed by ±10% to generate upper and lower distance bounds to account for experimental and simulation uncertainties (Table S3). Notably, **6** exhibited a double set of amide ^1^H chemical shift resonances (Table S1) in slow exchange on the NMR timescale, suggesting the presence of a second conformational population. However, the resonances of the corresponding side chains were below detection; thus, only the set of NOEs deriving from the prevailing population were considered for structure calculation.

### Replica-Averaged Molecular Dynamics

The distances and ^3^*J* scalar coupling data from NMR experiments were incorporated in the molecular dynamics framework as structural restraints averaged over 10 parallel replicas of the system, starting from randomly generated conformations. The simulations were performed using the GROMACS 2018.8^65^ code patched with PLUMED 2.5.3.^66,67^ The peptide was built with the Maestro Suite 2019^68^ and then solvated in a 12 Å layer cubic DMSO box. The ff14SB^69^ Amber force field was used to parametrize the peptide, whereas the parameters for the solvent box were taken from a previous work by Fox and Kollman.^70^ Atom types and bonded parameters for the non-natural Chg amino acid were taken by homology from the Amber force field, while its atomic partial charges were predicted using the two-stage restrained electrostatic potential (RESP)^71^ fitting procedure implemented in Antechamber.^72^ Prior to the RESP fitting, the electrostatic potentials (ESPs) were computed with the aid of the quantomechanical package Gaussian16.^73^ A double-step geometry optimization procedure at Hartree–Fock level of theory was employed: a preliminary calculation with the 3-21G basis set, followed by an accurate refinement with the 6-31G* basis set, after which the ESPs were computed. The topology files of the systems were generated with the tleap program of AmbertTools19 and then converted into the Gromacs format with the ParmEd tool. During the simulations, a time step of 2 fs was employed, while the bonds were constrained using the noniterative LINCS algorithm.^74^ A cutoff of 12 Å was chosen for the evaluation of the short-range nonbonded interactions, whereas the long-range electrostatic ones were treated using the particle mesh Ewald^75^ method, using a 1.0 Å grid spacing in periodic boundary conditions. The system first underwent 10 000 steps of steepest descent energy minimization. Then, the simulation box was equilibrated and heated up to 300 K, alternating NPT and NVT cycles with the Berendsen^76^ coupling bath and barostat. Finally, 500 ns of long production runs were performed for each replica in the NPT ensemble, resulting in a total simulation time of 5 μs. During the production runs, pressure of 1 atm and temperature of 300 K were kept constant using the stochastic velocity rescaling^77^ and the Parrinello–Rahman^78^ algorithms, respectively. Finally, the trajectories were clustered based on the peptide backbone root-mean-square deviation (rmsd), and the centroid of the largest population was selected as the representative structure of the NMR ensemble.

### Molecular Dockings

The NMR-predicted conformation of **6** was docked in the crystal structure of either αvβ6 or αvβ8 receptor in complex with proTGF-β (PDB code: 5FFO and 6OM2, respectively);^56,57^ the cyclic peptide backbone was treated as rigid, whereas the side chains were kept flexible. The peptide and the receptors were prepared with the aid of the Protein Preparation Wizard tool as in previous papers.^79,80^ Missing hydrogen atoms were added and water molecules were deleted from the receptor structure. The co-crystalized Mg^2+^, Mn^2+^, and Ca^2+^ divalent cations at the protein MIDAS, adjacent to MIDAS (only in αvβ6), and LIMBS sites were retained and treated using the default parameters. The side chain ionization and tautomeric states were predicted using Epik.^81,82^ Prior to docking, the receptor was refined optimizing its hydrogen-bonding network and minimizing the position of the hydrogens. As for the grid generation, a virtual box of 25 Å × 25 Å × 25 Å, centered on the integrin-binding site, was computed through the Receptor Grid Generator tool of Glide 8.5.^83,84^ Docking calculations were performed using the Glide SP-peptide default parameters and the OPLS3e force field.^85^ Finally, the best ranked pose for each system was selected (docking scores: −7.855 and −8.320 for the predicted **6**/αvβ6 and **6**/αvβ8 complexes, respectively).

### Cells, Viruses, Plasmid, Antibody, and Soluble Proteins

293T (constitutively expressing both the αvβ6 and αvβ8 integrins at low levels) and J cells (derivatives of BHK-TK2 cells lacking any HSV receptor)^55^ were grown in Dulbecco’s modified Eagle’s medium (DMEM) containing 5% fetal bovine serum (FBS). R8102, a HSV-1 recombinant carrying LacZ under the control of the α27 promoter55, and K26GFP, a HSV-1 recombinant expressing green fluorescent protein (GFP) and plasmids encoding nectin1, αvβ6, and -β8 integrin, have been described in previous studies.^55,56^ GFR2Δ (denoted as Erb-2) carries the extracellular domain and transmembrane (TM) sequences of rat HER-2/neu (nucleotides 25–2096) (GenBank accession number NM_017003) and is deleted of the tyrosine kinase domain was described.^80^ R1.302, a nectin1-neutralizing monoclonal antibody (mAb), was given by Lopez. Human integrin β8-APC-conjugated antibody (FAB4775A) and human integrin β6-APC-conjugated antibody (FAB4155A) were purchased from R&D Systems a Biotechne brand. APC mouse IgG1 κ (clone MOPC-21) was purchased from Becton Dickinson Pharmingen. gH soluble form (gH_solST_) and gB soluble form (gB_solST_) expressing the One-STrEP tag epitope (ST) have been previously described.^38^ PE-conjugated mAb to the One-STrEP tag (Strep-Tactin) was purchased from IBA Solutions for Life Sciences.

### Infection Neutralization Assays

Lyophilized peptide **1**, **6**, or cilengitide were dissolved in DMEM without serum at a 10 mM concentration; the solution was adjusted to a neutral pH by addition of 1 M Tris–HCl, pH 10. The experiments were performed using 293T cells in 96-well plates with extracellular virions of R8102 at an input multiplicity of infection of 5 PFU/cell. Cells were exposed to increasing concentrations of the peptides for 1 h at 37 °C, and the viral inoculum was added in the presence of peptides for 90 min at 37 °C. After the removal of the inoculum and rinsing with DMEM containing 1% FBS, the cells were incubated without peptides for 8 h. The same protocol was followed for R1.302 mAb that was used as a positive control. The infection was quantified as β-Gal activity using *o*-nitrophenyl-d-galactopyranoside (ONPG) as a substrate.^86^

In other experiments, J cells were transfected with low amounts of plasmids encoding nectin1 (75 ng of DNA/24 well), plus or minus plasmids encoding αv + β6 or αv + β8 integrins (300 ng of DNA/24 well) or together by means of Lipofect 2000 (Life Technologies). The total amount of transfected plasmid DNA was made equal (675 ng/24 well) by the addition of Erb-2 plasmid DNA. Then, 48 h after transfection, cells were incubated with a single concentration of peptides (700 μM) for 1 h at 37 °C or with mAb R1.302 (700 μM) and infected with 1 PFU/cell of K26GFP for 90 min at 37 °C in the presence of peptides or antibody. Following infection, the unpenetrated virus was inactivated by means of an acid wash (40 mM citric acid, 10 mM KCl, 135 mM NaCl, pH 3),^87^ and the cells were incubated for a further 16 h in the absence of the peptides or antibody. The extent of infection was assessed through EGFP expression.

### Integrin Internalization and the gH Binding Assay

J cells were transfected with low amounts of plasmids encoding nectin1 (75 ng of DNA/24 well), plus plasmids encoding αv + β6 and αv + β8 integrins (300 ng of DNA/24 well) by means of Lipofect 2000 (Life Technologies). Then, 48 h after transfection, the cells were incubated with a single concentration of peptides (700 μM) for 1 h at 37 °C. At the end of the treatment with the peptides, half of the sample was used to visualize the expression of integrins β6 and β8 or of nectin1 used as a control in a flow cytometry assay. Cells derived from samples treated or not treated with peptides were incubated for 1 h at 4 °C with the following mAbs: FAB4155A to detect integrin β6, FAB4775A to detect integrin β8 and R1.302 to detect nectin1. To visualize nectin1 the samples incubated with R1.302 were washed and subsequently incubated 1 h at 4 °C with the APC mouse secondary antibody. Cytofluorimetric analyses were performed using an AccuriC6 flow cytometer (Becton Dickinson). A least 10 000 events were acquired for each sample.

The remaining half of the samples were used for the gH binding assay. Cells were incubated for 1 h at 4 °C with 2 μM gH_solST_ in 100 μL of DMEM containing 5% FBS and 30 mM *N*-(2-hydroxyethyl)piperazine-*N*′-ethanesulfonic acid (HEPES), washed three times with the same buffer, and further incubated for 1 h a 4 °C with PE-conjugated mAb to the One-STrEP tag (Strep-Tactin) for detection. Control cells were incubated with 2 μM gB_solST_ in 100 μL of the same buffer used for gH_solST_ and the binding of soluble protein was detected as described for the gH binding assay. Cells were analyzed by cytofluorimetric analyses using an AccuriC6 flow cytometer (Becton Dickinson). A least 10 000 events were acquired for each sample.

### Flow Cytometry and Microphotography

The inhibition of HSV infection by **1**, **6**, cilengitide, or mAb R1.302 was quantified through enhanced green fluorescent protein (EGFP) expression from the K26GFP virus. Cytofluorimetric analyses were performed using an AccuriC6 flow cytometer (Becton Dickinson). A least 10 000 events were acquired for each sample. Microphotography of the inhibition experiment was conducted using a Nikon Eclipse Ni microscope.

## Supporting Information Available

Analytical data, HPLC chromatograms, and HRMS spectra of **2**–**6**; NMR data (chemical shifts, ^3^*J* coupling assignments, temperature coefficients, and NOE-derived distance restraints) and spectra of **6**; superposition of the NMR-derived structures of **1** and **6**; and superposition of the **1**/αvβ6 docking complex with αvβ8 (PDF)
Molecular formula strings (CSV)
PDB file of the **6**/αvβ6 docking complex (PDB)
PDB file of **the 6**/αvβ8 docking complex (PDB)

## Supplementary Material

jm1c00533_si_002.csv

jm1c00533_si_003.pdb

jm1c00533_si_004.pdb

## Abbreviations

APC: allophycocyanin
DIPEA: *N*,*N*-diisopropylethylamine
DMBA: dimethylbarbituric acid
DMEM: Dulbecco’s modified Eagle’s medium
DMS: dimethylsulfate
DSS: 2,2-dimethyl-2-silapentane-5-sulfonate
ECM: extracellular matrix
EGFP: enhanced green fluorescent protein
FBS: fetal bovine serum
GFP: green fluorescent protein
HBTU: *o*-benzotriazole-*N*,*N*,*N′*,*N′*-tetra-methyluroniumhexafluorophosphate
HOAt: 1-hydroxy-7-azabenzotriazole
HOBt: 1-hydroxybenzotriazole hydrate
HVEM: herpesvirus entry mediator
FMDV: foot-and-mouth disease virus
IPF: idiopathic pulmonary fibrosis
LAP: latency-associated peptide
mAbs: monoclonal antibodies
MFI: mean fluorescence intensity
NMP: *N*-methylpirrolidone
*o*NBS-amide: *ortho*-nitrobenzensulfonylamide
*o*NBS-Cl: *ortho*-nitrobenzenesulfonyl chloride
ONPG: *o*-nitrophenyl-d-galactopyranoside
PBS: phosphate-buffered saline tablets
PyAOP: (1*H*-7-azabenzotriazol-1-yl-oxy)tris-pyrrolidinophosphonium hexafluorophosphate
SD: standard deviation
TEA: triethylamine
TFA: trifluoroacetic acid
TGF-β: transforming growth factor
TIS: triisopropylsilane
TM: transmembrane
TMB: tetramethylbenzidine
TOCSY: total correlation spectroscopy
US-SPPS: ultrasound-assisted solid-phase peptide synthesis protocol
