# Supplementary material for: Halting the Spread of Herpes Simplex Virus-1: The Discovery of an Effective Dual αvβ6/αvβ8 Integrin Ligand
Source: J Med Chem. 2021 May 7;64(10):6972–84. doi: 10.1021/acs.jmedchem.1c00533 (PMC8279406; doi:10.1021/acs.jmedchem.1c00533)
Supplement: Supplementary file 1 — jm1c00533_si_001.pdf [file jm1c00533_si_001.pdf]

SUPPORTING INFORMATION

Halting the Spread of Herpes Simplex Virus-1: Discovery of an Effective Dual αvβ6/αvβ8 Integrin Ligand

Stefano Tomassi,[1,‡] Vincenzo Maria D'Amore,[1,‡] Francesco Saverio Di Leva,[1,] Andrea Vannini,[2] Giacomo Quilici,[3] Michael Weinmüller,[4] Florian Reichart,[4] Jussara Amato,[1] Barbara Romano,[1] Angelo Antonio Izzo,[1] Salvatore Di Maro,[5,*] Ettore Novellino,[1,6] Giovanna Musco,[3] Tatiana Gianni,[2] Horst Kessler,[4] Luciana Marinelli[1,*]*

[1]Dipartimento di Farmacia, Università degli Studi di Napoli "Federico II", Via D. Montesano 49, 80131 Naples, Italy.

[2]Department of Experimental, Diagnostic and Specialty Medicine, University of Bologna, 40126 Bologna, Italy.

[3]Biomolecular NMR Unit c/o IRCCS S. Raffaele, Via Olgettina 58, 20132 Milano, Italy.

[4]Institute for Advanced Study and Center of Integrated Protein Science (CIPSM), Department of Chemistry, Technische Universität München, Lichtenbergstraße 4, 85748 Garching, Germany.

[5]DiSTABiF, University of Campania "Luigi Vanvitelli", Via Vivaldi 43, 81100 Caserta, Italy.

[6]Facoltà di Medicina e Chirurgia, Università Cattolica del Sacro Cuore, Largo Francesco Vito, 1, 00168 Roma, Italy.

[‡]These authors contributed equally

*(F.S.D.L.) Phone: +39 0823 274 579. E-mail: francesco.dileva@unina.it

*(S.D.M) Phone: +39 0823 274 579. E-mail: salvatore.dimaro@unicampania.it

*(L.M.) Phone: +39 081 679 799. E-mail: lmarinel@unina.it

TABLE OF CONTENTS

Analytical data of compounds 2-6……………………………………..……....… **p. S2**

Scheme S1-S4: Synthetic procedures for compounds **2, 3, 5** and **6**.………..………… **p. S3-4**

Table S1: NMR chemical shifts and ^{3}J coupling assignment of **6**……….................... **p. S5**

Table S2: Temperature coefficient data of **6** in d_6-DMSO……………....……..…..... **p. S5**

Table S3: NOE derived distance restraints for **6**…….………….…………….……… **p. S6-7**

Table S4: Molecular Formula Strings of RTDLDSLRT, cilengitide and **1-6**………… **p. S7**

Figure S1: Arg^{1}-CO/Chg^{4}-NH interatomic distance over RAMD simulation on **6**…… **p. S8**

Figure S2: Superimposition of the NMR-derived structures of **1** and **6**…….…….…… **p. S8**

Figure S3: Superimposition of the **1**/αvβ6 docking complex with αvβ8…….………. **p. S9**

Figure S4-S8: HPLC chromatograms of compounds **2-6**………………..…………… **p. S9-11**

Figure S9-13: HRMS spectra of compounds **2-6**……..…………..…….……..… **p. S11-12**

Figure S14: ^{1}H-1D NMR spectrum of **6**…………………..……..……………....…... **p. S13**

Figure S15: ^{1}H-^{1}H TOCSY spectrum of **6**………………..……………………..… **p. S13**

Figure S16: ^{1}H-^{1}H NOESY spectrum of **6**……..………..…..……………………... **p. S14**

Figure S17: ^{1}H-^{1}H ROESY spectrum of **6**…………………...……………………... **p. S14**

Figure S18: ^{1}H-^{13}C HSQC spectrum of **6**…..………………….......................... **p. S15**

Supplementary References: ……….. **p. S15**

Analytical data of compounds 2-6:

Peptide [(*N*Me)Arg-Gly-Asp-Chg-Glu]CONH$_2$ (2):

23 mg, crude yield: 42 %, purity: ≥ 95%, t_R 12.31 min, (analytical HPLC, 10 to 90% acetonitrile (0.1% TFA) in water (0.1% TFA) over 20 min, flow rate of 1.0 mL/min); HRMS (ESI-MS): calculated: 610.33074 for $C_{26}H_{44}N_9O_8$ $[M+H]^+$, found: 610.33191. Calculated: 632.31323 for $C_{26}H_{43}N_9NaO_8$ $[M+Na]^+$, found: 632.31238.

Peptide [Arg-(*N*Me)Gly-Asp-Chg-Glu]CONH$_2$ (3):

35 mg, crude yield: 65 %, purity: ≥ 95%, t_R 12.74 min, (analytical HPLC, 10 to 90% acetonitrile (0.1% TFA) in water (0.1% TFA) over 20 min, flow rate of 1.0 mL/min); HRMS (ESI-MS): calculated: 610.33074 for $C_{26}H_{44}N_9O_8$ $[M+H]^+$, found: 610.33044. Calculated: 632.31323 for $C_{26}H_{43}N_9NaO_8$ $[M+Na]^+$, found: 632.31183

Peptide [Arg-Gly-(*N*Me)Asp-Chg-Glu]CONH$_2$ (4)

28 mg, crude yield: 51 %, purity: ≥ 95%, t_R 11.40 min, (analytical HPLC, 10 to 90% acetonitrile (0.1% TFA) in water (0.1% TFA) over 20 min, flow rate of 1.0 mL/min); HRMS (ESI-MS): calculated: 610.33074 for $C_{26}H_{44}N_9O_8$ $[M+H]^+$, found: 610.33246. Calculated: 632.31323 for $C_{26}H_{43}N_9NaO_8$ $[M+Na]^+$, found: 632.31219.

Peptide [Arg-Gly-Asp-(*N*Me)Chg-Glu]CONH$_2$ (5):

29 mg, crude yield: 53 %, purity: ≥ 95%, t_R 11.57 min, (analytical HPLC, 10 to 90% acetonitrile (0.1% TFA) in water (0.1% TFA) over 20 min, flow rate of 1.0 mL/min); HRMS (ESI-MS): calculated: 610.33074 for $C_{26}H_{44}N_9O_8$ $[M+H]^+$, found: 610.33197. Calculated: 632.31323 for $C_{26}H_{43}N_9NaO_8$ $[M+Na]^+$, found: 632.31165.

Peptide [Arg-Gly-Asp-Chg-(*N*Me)Glu]CONH$_2$ (6)

28 mg, crude yield: 49 %, purity: ≥ 95%, t_R 12.11 min, (analytical HPLC, 10 to 90% acetonitrile (0.1% TFA) in water (0.1% TFA) over 20 min, flow rate of 1.0 mL/min); HRMS (ESI-MS): calculated: 610.33074 for $C_{26}H_{44}N_9O_8$ $[M+H]^+$, found: 610.33215. Calculated: 632.31323 for $C_{26}H_{43}N_9NaO_8$ $[M+Na]^+$, found: 632.31195.

Scheme S1. Synthetic strategy for [(*N*Me)Arg-Gly-Asp-Chg-Glu]CONH2 (**2**):

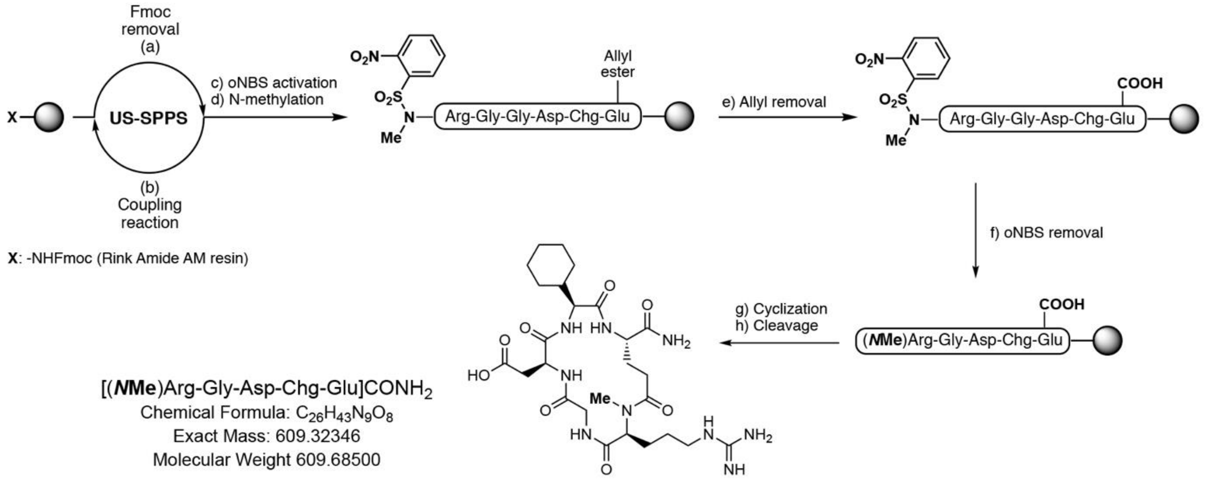

a) Piperidine 20% in DMF, 2 × 1 min, US irradiation; b) Fmoc-AA-OH, HBTU, HOBt, DIPEA, DMF, 5 min, US irradiation; c) *o*NBS chloride, TEA, dry DCM, rt, 2 × 30 min; d) Dimethylsulfate, DBU, dry NMP, room temperature, 2 × 30 min; e) $Pd(PPh_3)_4$, DMBA, DCM/DMF 2:1, 2 × 60 min; f) Mercaptoethanol, DBU, dry DMF, room temperature, 3 × 15 min; g) PyAOP, HOAt, DIPEA, DMF, room temperature, 6 h; h) TFA/TIS 95:5, room temperature, 3 h.

Scheme S2. Synthetic strategy for [Arg-(*N*Me)Gly-Asp-Chg-Glu]CONH2 (**3**):

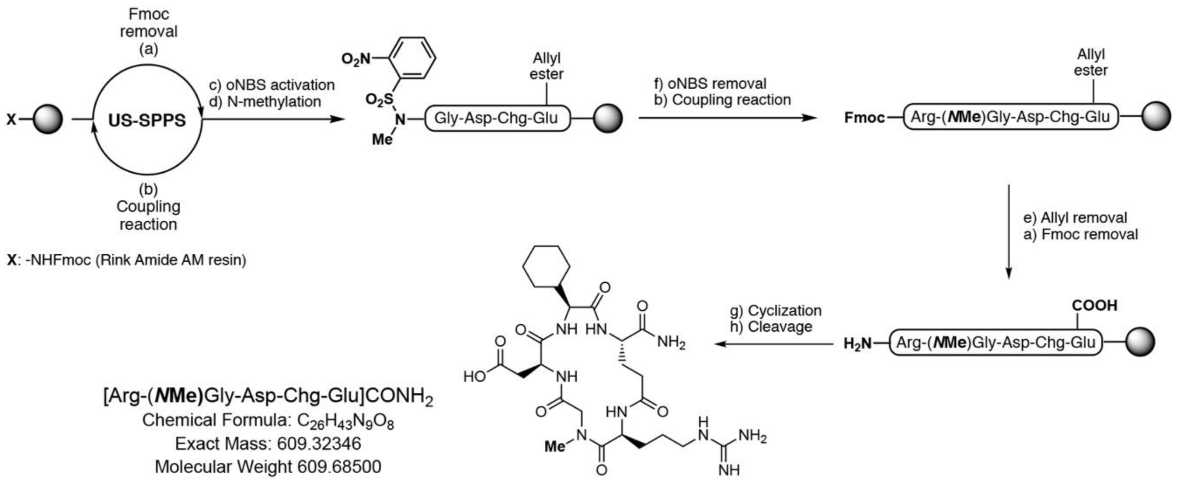

a) Piperidine 20% in DMF, 2 × 1 min, US irradiation; b) Fmoc-AA-OH, HBTU, HOBt, DIPEA, DMF, 5 min, US irradiation; c) *o*NBS chloride, TEA, dry DCM, rt, 2 × 30 min; d) Dimethylsulfate, DBU, dry NMP, room temperature, 2 × 30 min; e) $Pd(PPh_3)_4$, DMBA, DCM/DMF 2:1, 2 × 60 min; f) Mercaptoethanol, DBU, dry DMF, room temperature, 3 × 15 min; g) PyAOP, HOAt, DIPEA, DMF, room temperature, 6 h; h) TFA/TIS 95:5, room temperature, 3 h.

Scheme S3. Synthetic strategy for [Arg-Gly-Asp-(*N*Me)Chg-Glu]$CONH_2$ (**5**):

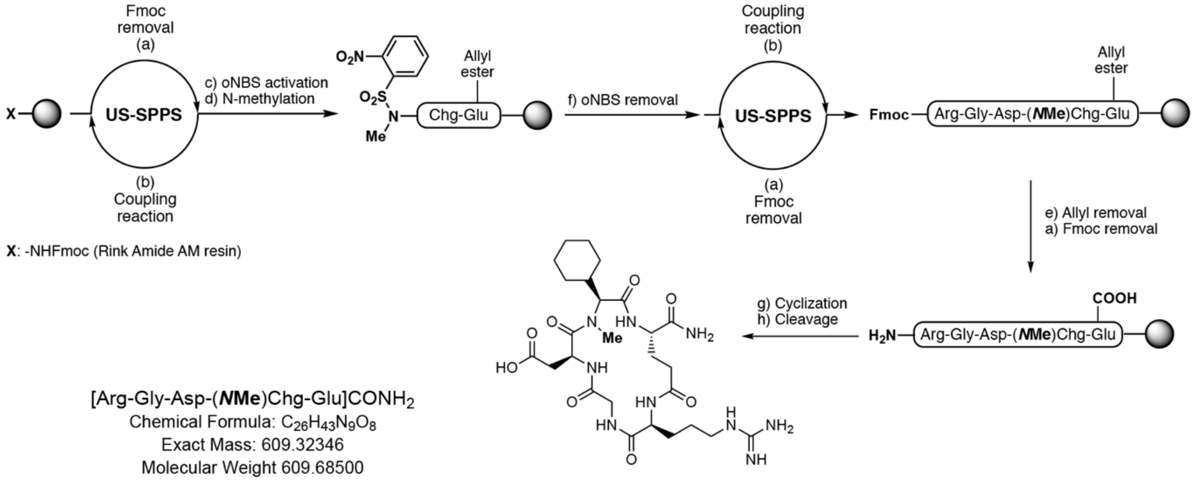

a) Piperidine 20% in DMF, 2 × 1 min, US irradiation; b) Fmoc-AA-OH, HBTU, HOBt, DIPEA, DMF, 5 min, US irradiation; c) *o*NBS chloride, TEA, dry DCM, rt, 2 × 30 min; d) Dimethylsulfate, DBU, dry NMP, room temperature, 2 × 30 min; e) $Pd(PPh_3)_4$, DMBA, DCM/DMF 2:1, 2 × 60 min; f) Mercaptoethanol, DBU, dry DMF, room temperature, 3 × 15 min; g) PyAOP, HOAt, DIPEA, DMF, room temperature, 6 h; h) TFA/TIS 95:5, room temperature, 3 h.

Scheme S4. Synthetic strategy for [Arg-Gly-Asp-Chg-(*N*Me)Glu]$CONH_2$ (**6**):

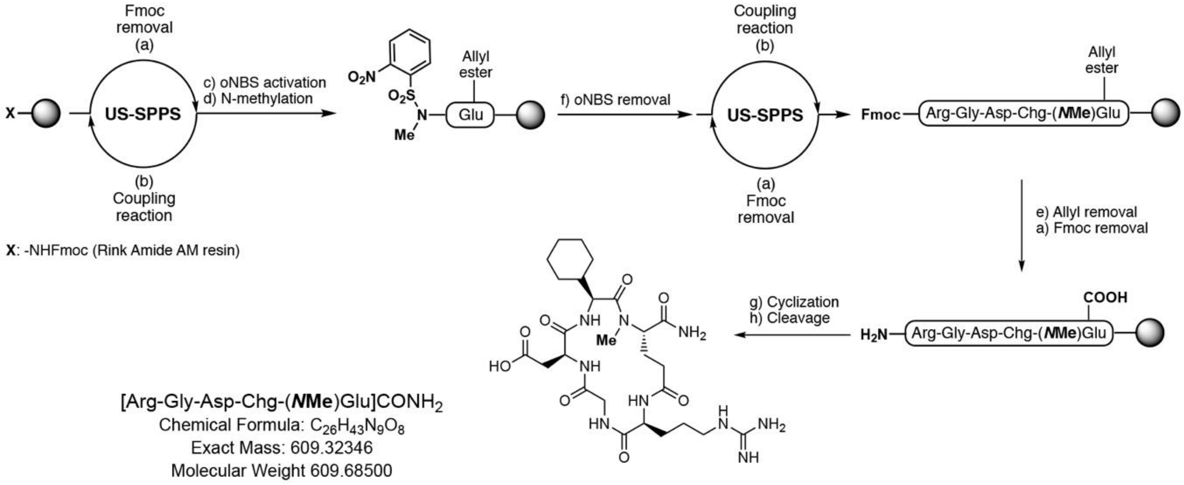

a) Piperidine 20% in DMF, 2 × 1 min, US irradiation; b) Fmoc-AA-OH, HBTU, HOBt, DIPEA, DMF, 5 min, US irradiation; c) *o*NBS chloride, TEA, dry DCM, rt, 2 × 30 min; d) Dimethylsulfate, DBU, dry NMP, room temperature, 2 × 30 min; e) $Pd(PPh_3)_4$, DMBA, DCM/DMF 2:1, 2 × 60 min; f) Mercaptoethanol, DBU, dry DMF, room temperature, 3 × 15 min; g) PyAOP, HOAt, DIPEA, DMF, room temperature, 6 h; h) TFA/TIS 95:5, room temperature, 3 h.

Table S1. $^3J_{HN\text{-}H\alpha}$ scalar couplings and chemical shifts (ppm) of **6**. Assignment was determined in d_6-DMSO at 298K. The experimental error estimated for the ^3J couplings is 0.1 Hz.

	$^3J_{HN\text{-}H\alpha}$		HN	α	β	γ	δ	ε	Me	1	2 (2')†	3 (3')‡	4‡
**Arg^1**	8.0	**^{1}H**	7.88<br>*(8.64)	4.19	1.51<br>1.70	1.43<br>1.48	3.08<br>3.08	7.45					
		**^{13}C**		55.77	30.70	28.42	43.60						
**Gly^2**	5.19<br>5.44	**^{1}H**	7.85<br>(8.27)	3.63<br>3.74									
		**^{13}C**		42.61									
**Asp^3**	7.1	**^{1}H**	7.94<br>(8.50)	4.27	2.76<br>2.76								
		**^{13}C**		53.85	38.78								
**Chg^4**	8.8	**^{1}H**	7.74<br>(7.38)	4.37						1.74	0.96a,1.61e<br>(0.87a,1.73e)	1.11a,1.62e<br>(1.12a,1.65e)	1.11,1.59e
		**^{13}C**		57.25						42.1	32.90<br>(29.06)	28.90<br>(28.90)	29.06
**(*N*Me) Glu^5**	7.6	**^{1}H**		4.85	1.73<br>1.94	2.09<br>2.17		7.11<br>7.19	2.80				
		**^{13}C**		59.04	26.69	35.20			33.84				

a: axial
e: equatorial
†: Assignment of ^{1}H-^{13}C 2 and 2' are not stereospecific
‡: Assignments of ^{1}H-^{13}C 3, 3' and 4 are ambiguous, i.e. the assignments can be swapped
*: chemical shifts in brackets correspond to amides of the second conformation in slow exchange on the NMR time scale with the amides of the predominant conformation.

Table S2. Temperature coefficient data of 6. ^{1}H-1D spectra were recorded varying the temperature from 285 K to 305 K in 5 K intervals, the temperature coefficients were calculated according to $(\Delta\delta*1000)/\Delta T$, where $\Delta\delta$ and ΔT are changes in chemical shift of the corresponding NH and the change in sample temperature, respectively.

NH Residue	Temperature Coefficient (ppb/K)
Arg^1	-3.2
Gly^2	-2.6
Asp^3	-1.6
Chg^4	-7.0

Table S3. NOE interactions and distance restraints used to derive the structure of **6**.

**Residue-1**	**Atom-1**	**Residue-2**	**Atom-2**	**NOE Lower Distance (nm)**	**NOE Upper Distance (nm)**
Arg^1	HN	Arg^1	Hα	0.23	0.28
Arg^1	Hβa	Arg^1	HN	0.23	0.28
Arg^1	Hβa	Arg^1	Hα	0.25	0.30
Arg^1	Hβa	Arg^1	Hε	0.28	0.35
Arg^1	Hβb	Arg^1	HN	0.25	0.31
Arg^1	Hβb	Arg^1	Hα	0.22	0.27
Arg^1	Hβb	Arg^1	Hδ*	0.31	0.38
Arg^1	Hγa	Arg^1	HN	0.25	0.31
Arg^1	Hγa	Arg^1	Hδ*	0.27	0.33
Arg^1	Hδ*	Arg^1	Hα	0.32	0.39
Arg^1	Hδ*	Arg^1	Hε	0.28	0.34
Gly^2	HN	Arg^1	Hα	0.20	0.24
Asp^3	HN	Gly^2	Hαa	0.22	0.27
Asp^3	HN	Gly^2	Hαb	0.19	0.24
Asp^3	Hα	Asp^3	HN	0.22	0.26
Asp^3	Hα	Chg^4	HN	0.23	0.28
Asp^3	Hβ*	Asp^3	HN	0.26	0.32
Asp^3	Hβ*	Asp^3	Hα	0.25	0.30
Asp^3	Hβ*	Chg^4	HN	0.31	0.38
Chg^4	HN	Chg^4	H2a	0.27	0.34
Chg^4	HN	Asp^3	HN	0.22	0.27
Chg^4	HN	Asp^3	Hα	0.24	0.29
Chg^4	HN	Asp^3	Hβ*	0.21	0.37
Chg^4	H1	Chg^4	Hα	0.26	0.32
Chg^4	H2a	Chg^4	Hα	0.25	0.31
Chg^4	H2e	Chg^4	Hα	0.26	0.32
Chg^4	H2’a	Chg^4	Hα	0.25	0.31
Chg^4	H2’a	(*N*Me)Glu^5	H*	0.38	0.46
Chg^4	H2’e	Chg^4	Hα	0.26	0.32
(*N*Me)Glu^5	Hα	(*N*Me)Glu^5	He21	0.29	0.35
(*N*Me)Glu^5	Hα	(*N*Me)Glu^5	He22	0.29	0.35
(*N*Me)Glu^5	Hα	Arg^1	HN	0.29	0.36
(*N*Me)Glu^5	Hβa	(*N*Me)Glu^5	Hα	0.26	0.31
(*N*Me)Glu^5	Hβa	(*N*Me)Glu^5	H*	0.27	0.33
(*N*Me)Glu^5	Hβb	(*N*Me)Glu^5	Hα	0.23	0.28
(*N*Me)Glu^5	Hβb	(*N*Me)Glu^5	H*	0.30	0.37
(*N*Me)Glu^5	Hβb	Arg^1	HN	0.28	0.34
(*N*Me)Glu^5	Hγa	(*N*Me)Glu^5	Hα	0.23	0.28
(*N*Me)Glu^5	Hγa	(*N*Me)Glu^5	H*	0.32	0.40
(*N*Me)Glu^5	Hγa	Arg^1	HN	0.2	0.27
(*N*Me)Glu^5	Hγb	(*N*Me)Glu^5	Hα	0.23	0.29
(*N*Me)Glu^5	Hγb	(*N*Me)Glu^5	H*	0.32	0.39

(*N*Me)Glu^5	Hγb	Arg^1	HN	0.22	0.27
(*N*Me)Glu^5	H*	(*N*Me)Glu^5	Hα	0.30	0.37
(*N*Me)Glu^5	H*	(*N*Me)Glu^5	He21	0.33	0.40
(*N*Me)Glu^5	H*	(*N*Me)Glu^5	He22	0.31	0.38
(*N*Me)Glu^5	H*	Asp^3	Hα	0.33	0.40
(*N*Me)Glu^5	H*	Chg^4	HN	0.34	0.41
(*N*Me)Glu^5	H*	Chg^4	Hα	0.26	0.32
(*N*Me)Glu^5	He21	(*N*Me)Glu^5	He22	0.12	0.15

*: pseudo atom correction applied, 0.06 and 0.1 nm for intra- and inter-residue respectively.[1]

Table S4. Molecular Formula Strings of RTDLDSLRT, cilengitide and **1**-**6**.

**Compound_ID**	**SMILES**
RTDLDSLRT	N[C@@H](CCCNC(N)=N)C(N[C@@H]([C@@H](C)O)C(N[C@@H](CC(O)=O)C(N[C@@H](CC(C)C)C(N[C@@H](CC(O)=O)C(N[C@@H](CO)C(N[C@@H](CC(C)C)C(N[C@@H](CCCNC(N)=N)C(N[C@@H]([C@@H](C)O)C(O)=O)=O)=O)=O)=O)=O)=O)=O)=O
cilengitide	O=C(O)C[C@@H](C(N[C@H](CC1=CC=CC=C1)C(N(C)[C@@H](C(C)C)C(N[C@@H](CCC/N=C(N)\N)C(NC2)=O)=O)=O)=O)NC2=O
**1**	O=C(NCC(N[C@@H](CC(O)=O)C(N[C@@H](C1CCCCC1)C(N[C@@H](CC2)C(N)=O)=O)=O)=O)[C@H](CCCNC(N)=N)NC2=O
**2**	O=C(NCC(N[C@@H](CC(O)=O)C(N[C@@H](C1CCCCC1)C(N[C@@H](CC2)C(N)=O)=O)=O)=O)[C@H](CCCNC(N)=N)N(C)C2=O
**3**	O=C(N(C)CC(N[C@@H](CC(O)=O)C(N[C@@H](C1CCCCC1)C(N[C@@H](CC2)C(N)=O)=O)=O)=O)[C@H](CCCNC(N)=N)NC2=O
**4**	O=C(NCC(N(C)[C@@H](CC(O)=O)C(N[C@@H](C1CCCCC1)C(N[C@@H](CC2)C(N)=O)=O)=O)=O)[C@H](CCCNC(N)=N)NC2=O
**5**	O=C(NCC(N[C@@H](CC(O)=O)C(N(C)[C@@H](C1CCCCC1)C(N[C@@H](CC2)C(N)=O)=O)=O)=O)[C@H](CCCNC(N)=N)NC2=O
**6**	O=C(NCC(N[C@@H](CC(O)=O)C(N[C@@H](C1CCCCC1)C(N(C)[C@@H](CC2)C(N)=O)=O)=O)=O)[C@H](CCCNC(N)=N)NC2=O

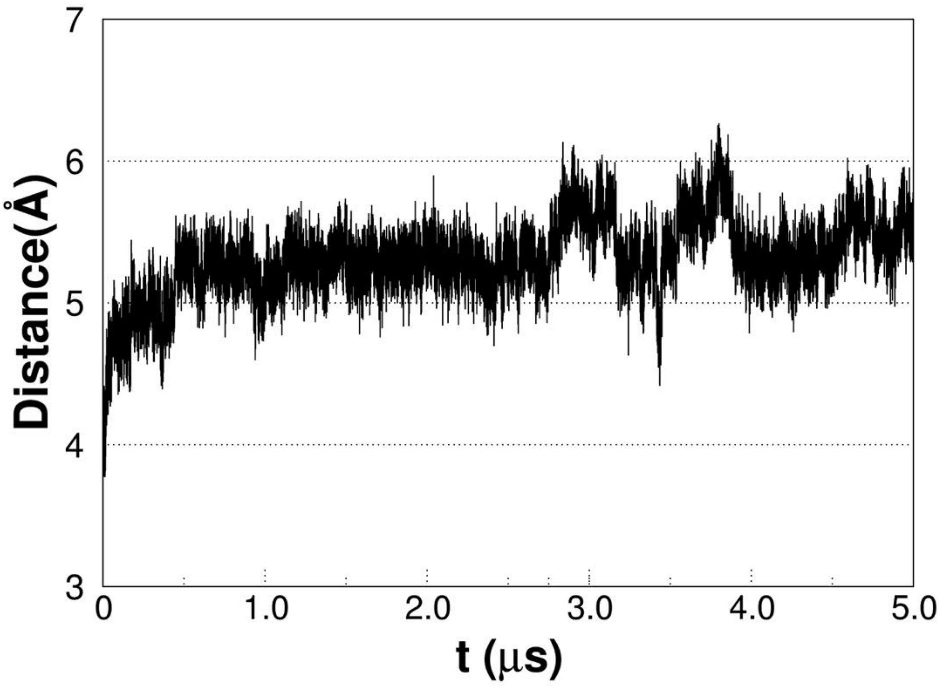

Figure S1. Ensemble-averaged interatomic distance between Arg^{1}-CO and Chg^{4}-NH over the RAMD simulation.

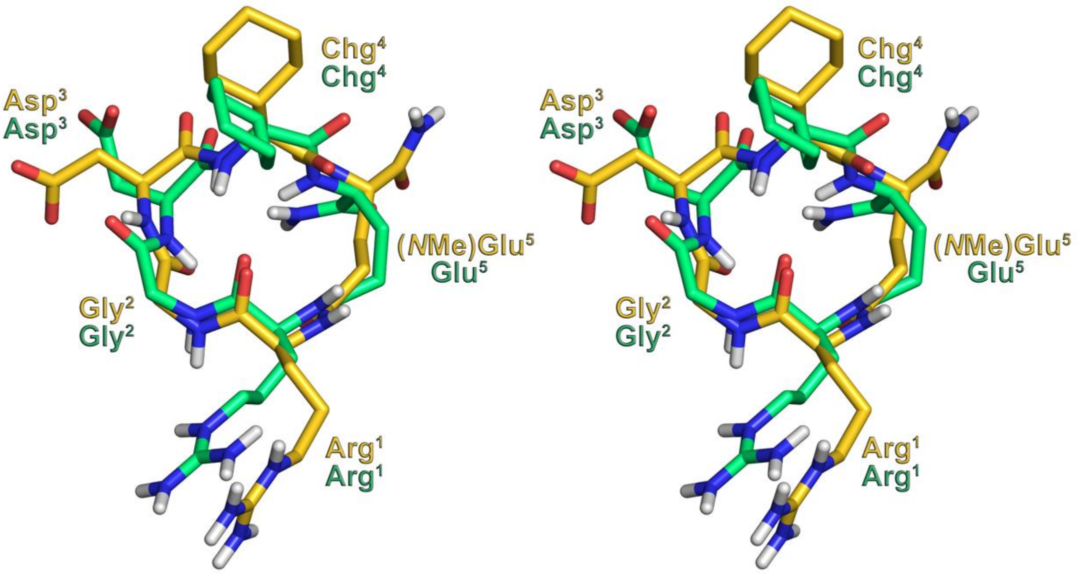

Figure S2. Stereo-view of the superimposition between the NMR-derived structures of **1** (green sticks) and **6** (gold sticks). Non-polar hydrogens are omitted for sake of clarity.

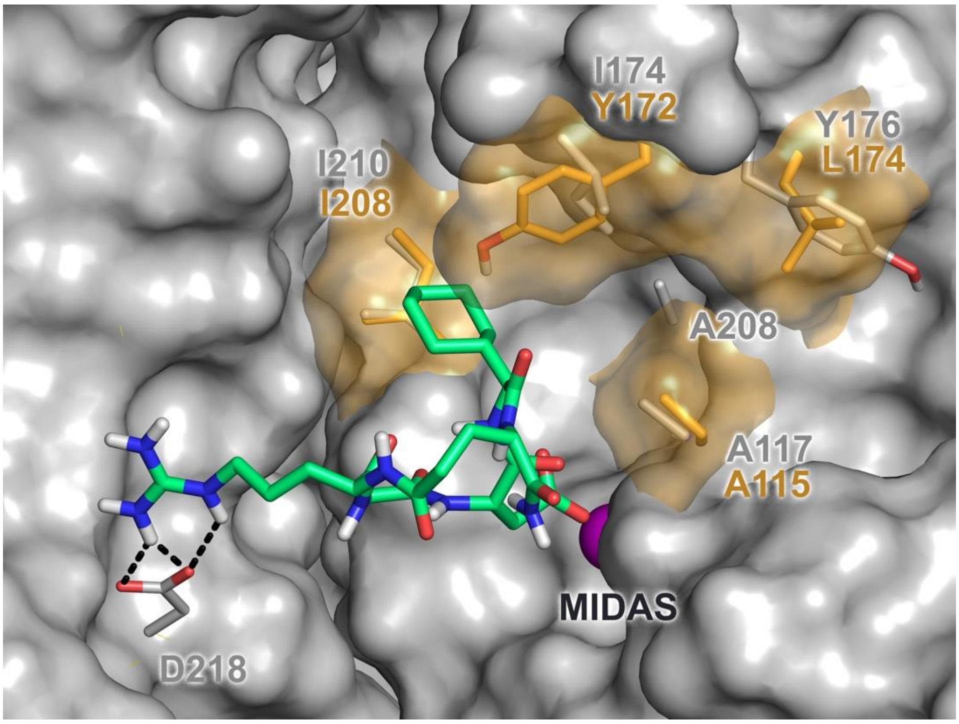

Figure S3. Superimposition of the **1**/αvβ6 (PDB code: 5FFO)[2] docking complex with the αvβ8 X-ray structure (PDB code: 6OM2)[3]. The peptide is shown as green sticks. The αvβ6 and αvβ8 receptors are represented as gray and orange surfaces, respectively. In both integrin subtypes, residues that are important to selectivity are highlighted as sticks and transparent surfaces for αvβ6 and αvβ8, respectively. The metal ion at MIDAS is shown as a purple sphere.

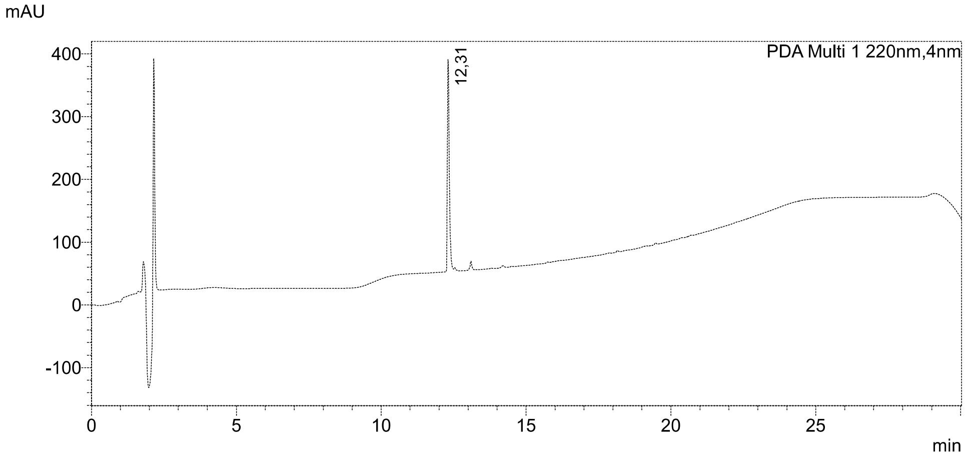

Figure S4. HPLC chromatogram of compound **2**.

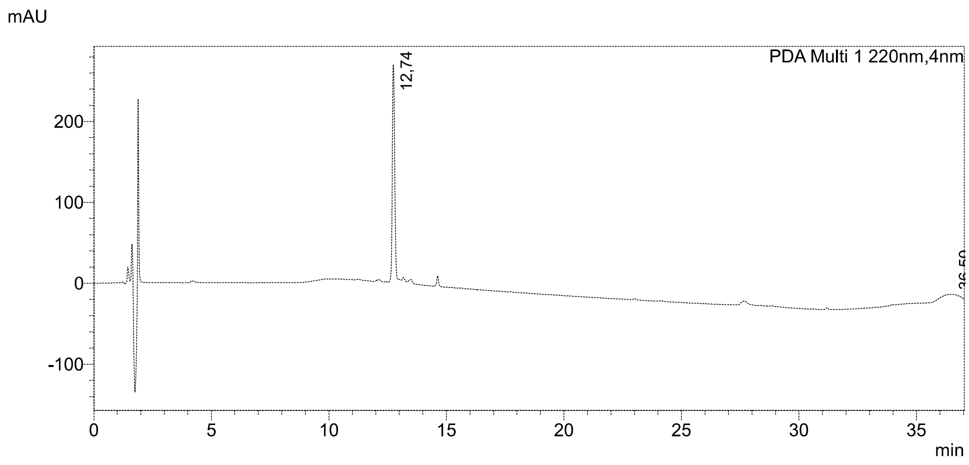

Figure S5. HPLC chromatogram of compound **3**.

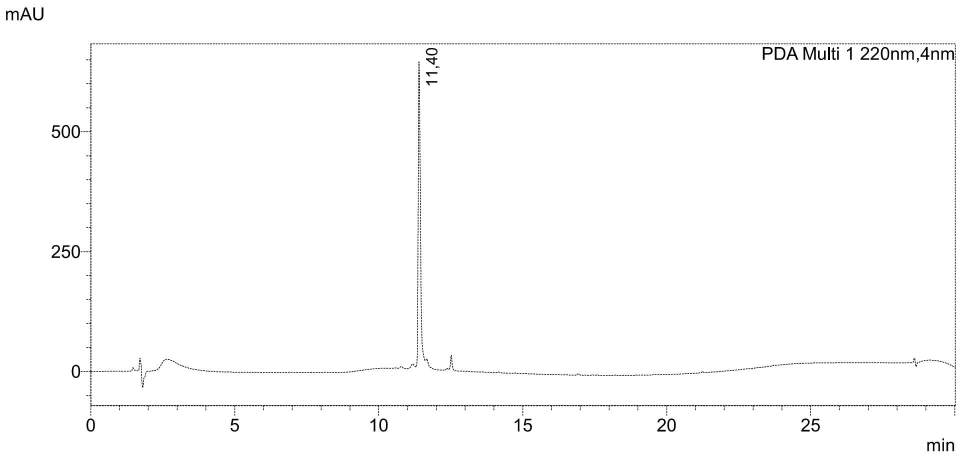

Figure S6. HPLC chromatogram of compound **4**.

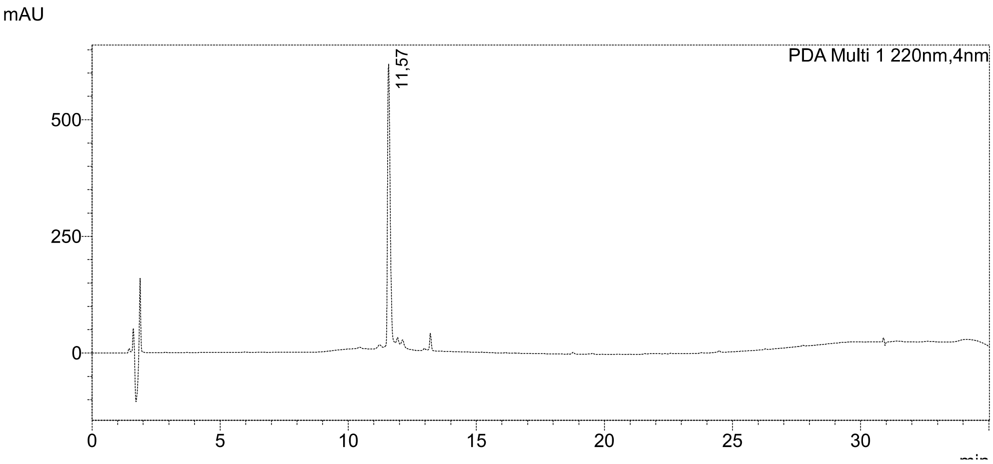

Figure S7. HPLC chromatogram of compound **5**.

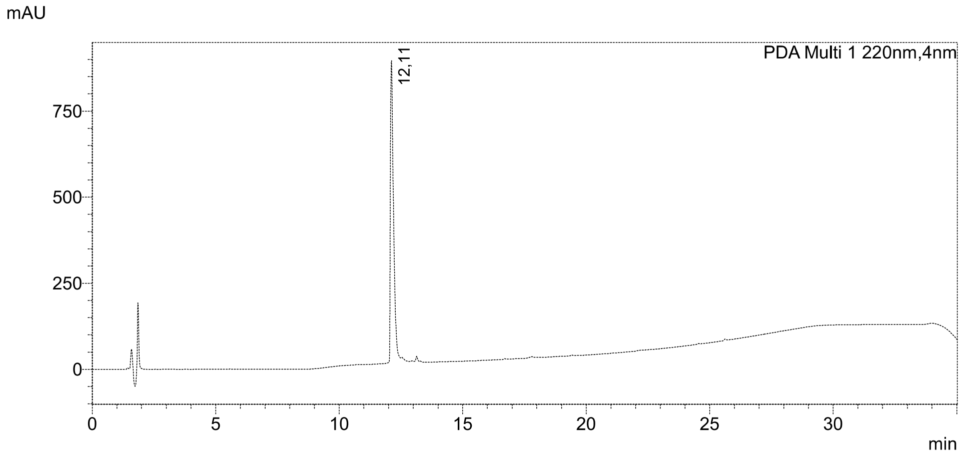

Figure S8. HPLC chromatogram of compound **6**.

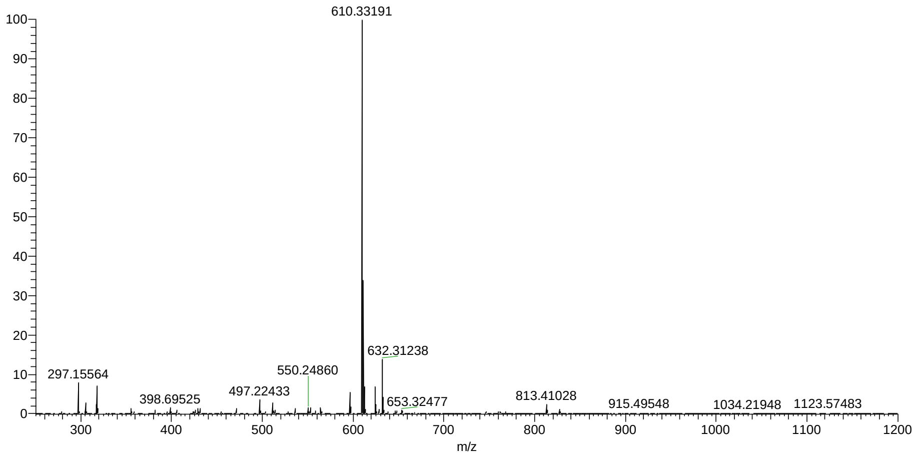

Figure S9. HRMS spectrum of compound **2**.

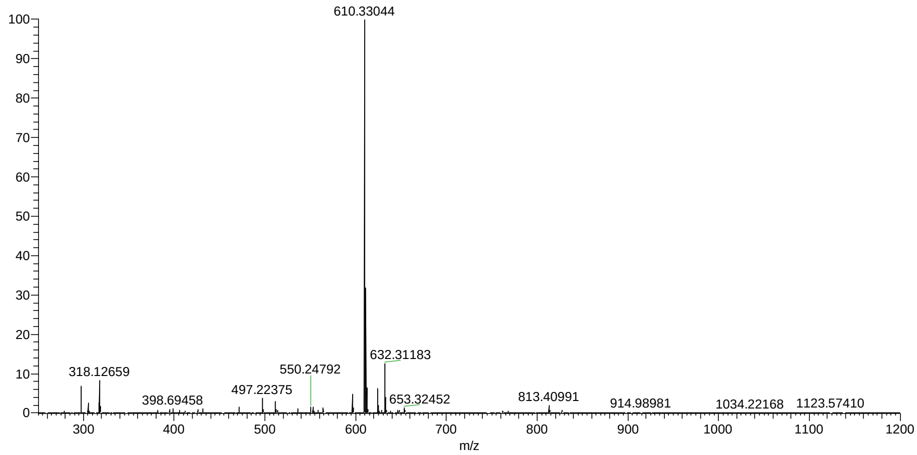

.

Figure S10. HRMS spectrum of compound **3**.

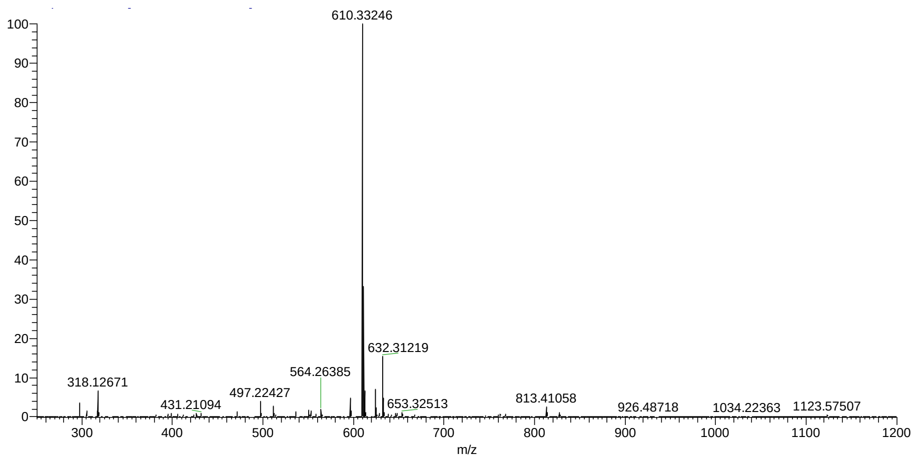

Figure S11. HRMS spectrum of compound **4**.

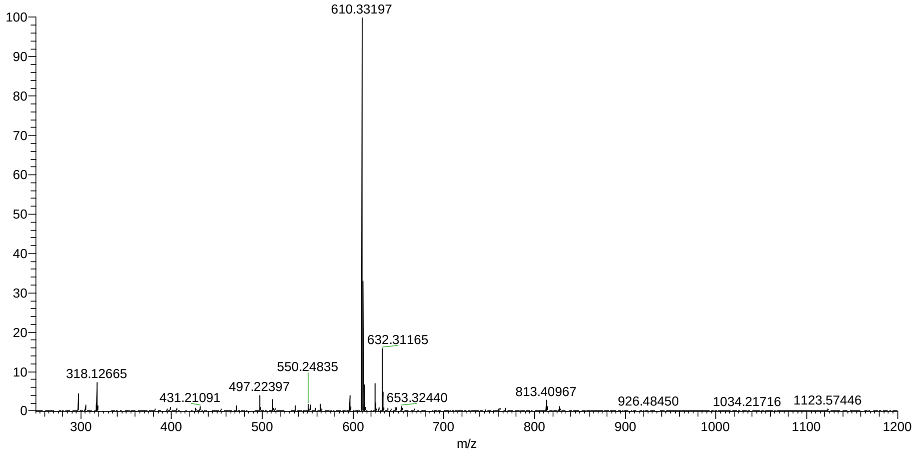

Figure S12. HRMS spectrum of compound **5**.

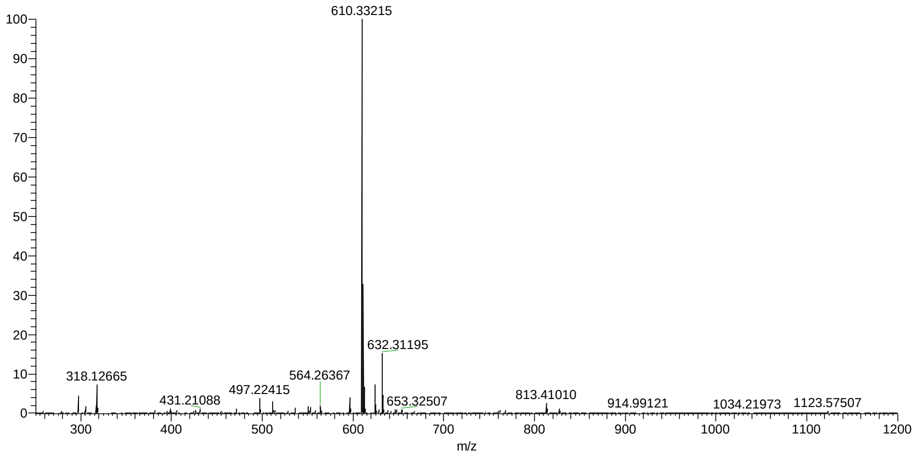

Figure S13. HRMS spectrum of compound **6**.

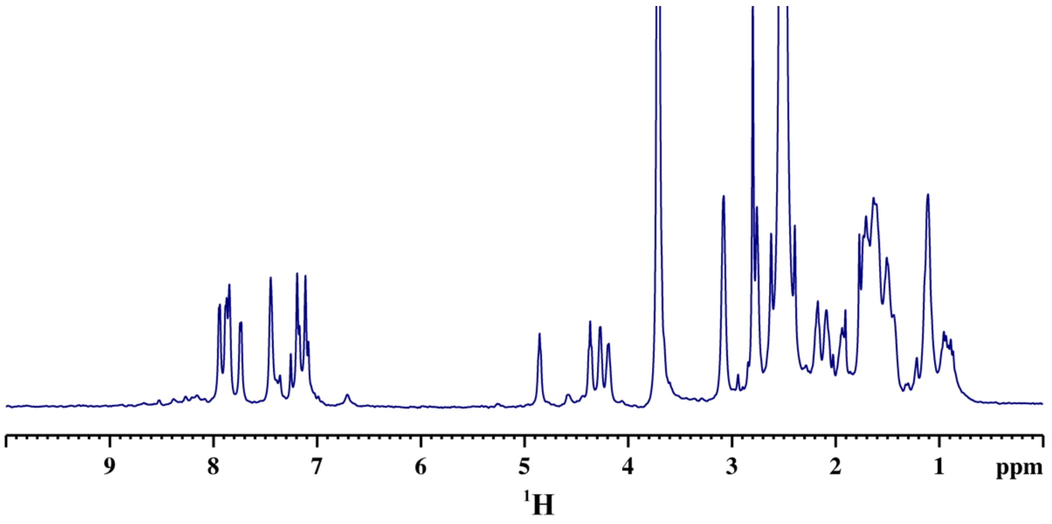

Figure S14. ^{1}H-1D NMR spectrum of **6**.

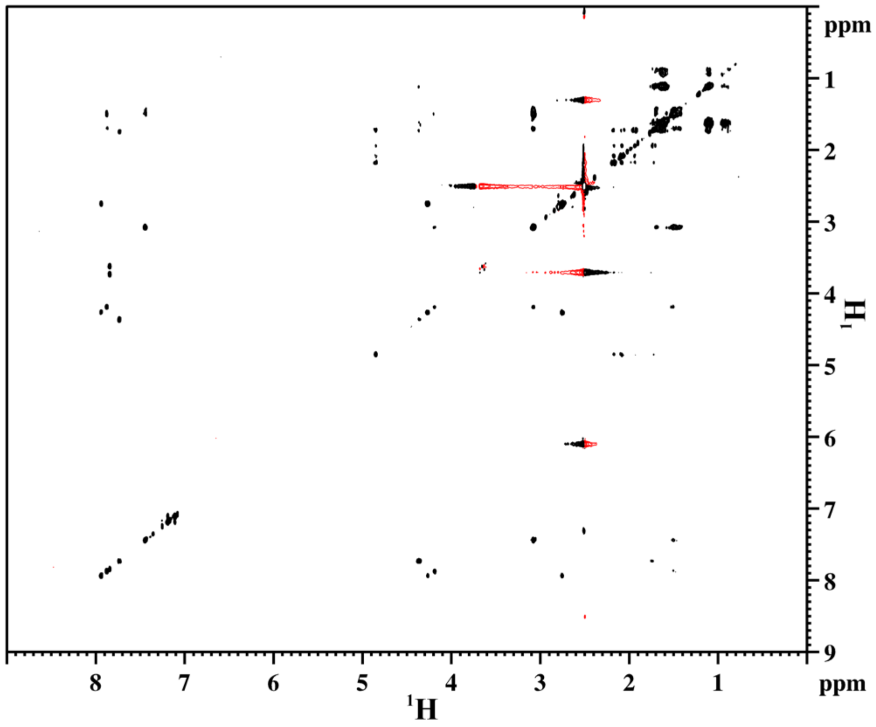

Figure S15. ^{1}H-^{1}H TOCSY spectrum of **6**.

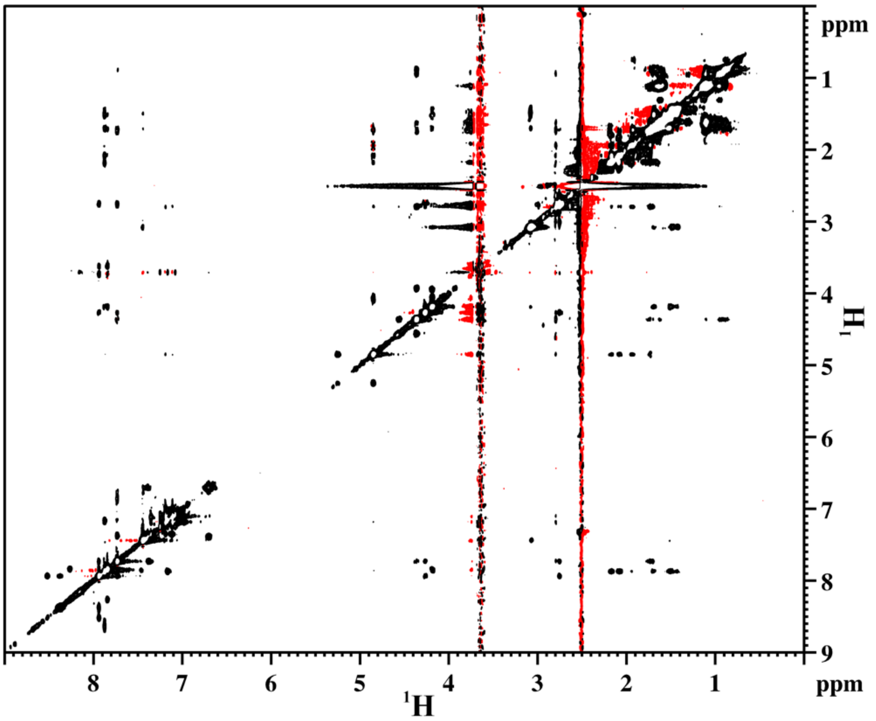

Figure S16. ^{1}H-^{1}H NOESY spectrum of **6**.

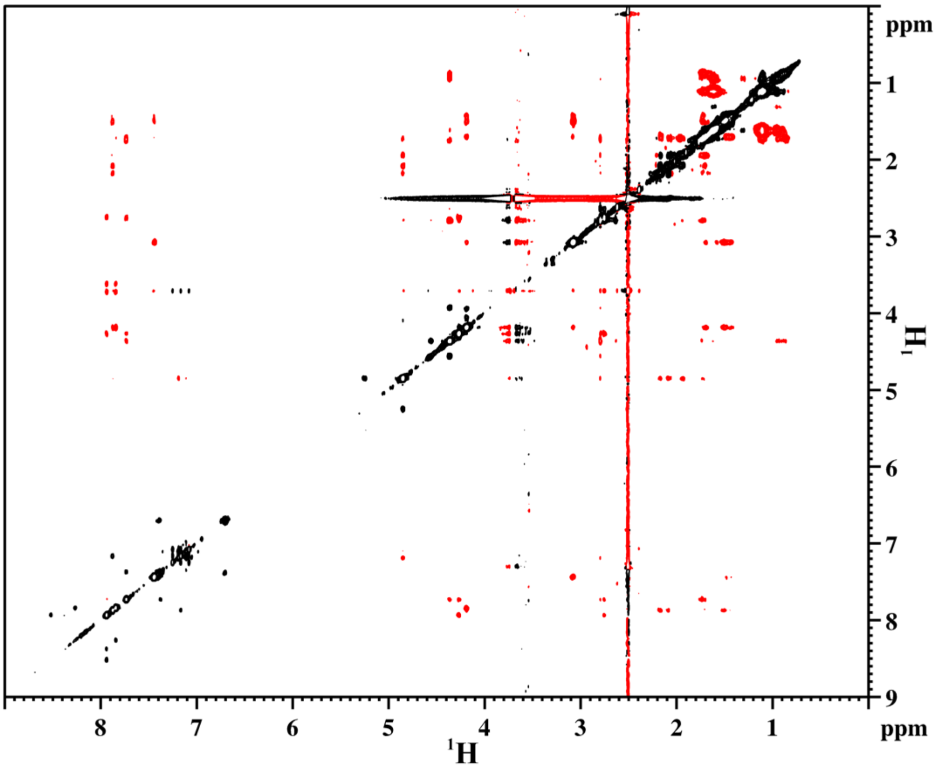

Figure S17. ^{1}H-^{1}H ROESY spectrum of **6**.

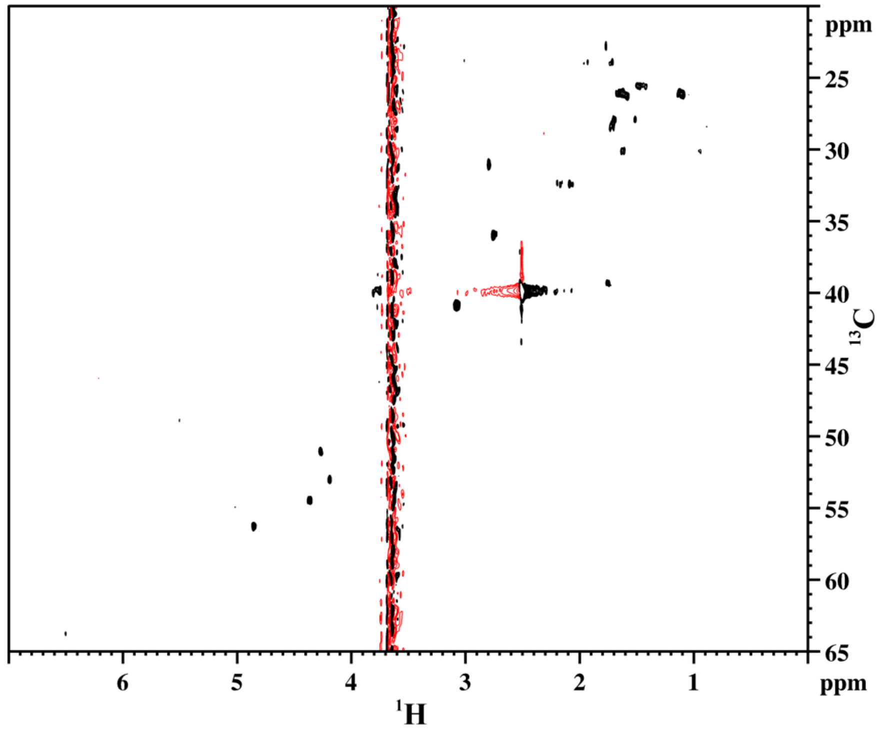

Figure S18. ^{1}H-^{13}C HSQC spectrum of **6.**

Supplementary References

(1) Braun, W.; Wider, G.; Lee, K. H.; Wüthrich, K. Conformation of Glucagon in a Lipid-Water Interphase by ^{1}H Nuclear Magnetic Resonance. *J. Mol. Biol.* **1983**, *169*, 921–948.

(2) Dong, X.; Zhao, B.; Iacob, R. E.; Zhu, J.; Koksal, A. C.; Lu, C.; Engen, J. R.; Springer, T. A. Force Interacts with Macromolecular Structure in Activation of TGF-β. *Nature* **2017**, *542*, 55–59.

(3) Wang, J.; Su, Y.; Iacob, R. E.; Engen, J. R.; Springer, T. A. General Structural Features That Regulate Integrin Affinity Revealed by Atypical αvβ8. *Nat. Commun.* **2019**, *10* , 1–13.
